# Microbial competition reduces metabolic interaction distances to the low µm-range

**DOI:** 10.1038/s41396-020-00806-9

**Published:** 2020-10-19

**Authors:** Rinke J. van Tatenhove-Pel, Tomaž Rijavec, Aleš Lapanje, Iris van Swam, Emile Zwering, Jhonatan A. Hernandez-Valdes, Oscar P. Kuipers, Cristian Picioreanu, Bas Teusink, Herwig Bachmann

**Affiliations:** 1 Systems Biology Lab, Amsterdam Institute of Molecular and Life Sciences, VU University Amsterdam, de Boelelaan 1108, 1081HV Amsterdam, The Netherlands; 2 Department of Environmental Sciences, Jožef Stefan Institute, Jamova cesta 39, 1000 Ljubljana, Slovenia; 3 Department of Molecular Genetics, Groningen Biomolecular Sciences and Biotechnology Institute, University of Groningen, Nijenborgh 7, 9747AG Groningen, The Netherlands; 4 Department of Biotechnology, Delft University of Technology, Van der Maasweg 9, 2629HZ Delft, The Netherlands; 5 NIZO Food Research, Kernhemseweg 2, 6718ZB Ede, The Netherlands

**Keywords:** Microbial ecology, Microbial communities

## Abstract

Metabolic interactions between cells affect microbial community compositions and hence their function in ecosystems. It is well-known that under competition for the exchanged metabolite, concentration gradients constrain the distances over which interactions can occur. However, interaction distances are typically quantified in two-dimensional systems or without accounting for competition or other metabolite-removal, conditions which may not very often match natural ecosystems. We here analyze the impact of cell-to-cell distance on unidirectional cross-feeding in a three-dimensional aqueous system with competition for the exchanged metabolite. Effective interaction distances were computed with a reaction-diffusion model and experimentally verified by growing a synthetic consortium of 1 µm-sized metabolite producer, receiver, and competitor cells in different spatial structures. We show that receivers cannot interact with producers located on average 15 µm away from them, as product concentration gradients flatten close to producer cells. We developed an aggregation protocol and varied the receiver cells’ product affinity, to show that within producer–receiver aggregates even low-affinity receiver cells could interact with producers. These results show that competition or other metabolite-removal of a public good in a three-dimensional system reduces metabolic interaction distances to the low µm-range, highlighting the importance of concentration gradients as physical constraint for cellular interactions.

## Introduction

Microbial interactions are observed in dense biofilms (0 µm between cells) as well as in oceans (>100 µm between cells), demonstrating that cells interact at various distances [1–4]. These interactions influence the selection pressure within an environment, and therefore affect the structure and evolution of microbial communities [5]. As these communities play an important role in many ecosystems, from global biogeochemical fluxes [6] to human health [7], understanding and controlling these interactions is of high importance.

Metabolites or signaling molecules involved in interactions can be exchanged via contact-dependent and contact-independent transfer mechanisms. Contact-dependent mechanisms require short cell-to-cell distances and use for instance direct contact between cells, vesicle chains, or nanotubes for exchange [5]. Contact-independent mechanisms require passive or active transport of the produced compound to the extracellular space, where it subsequently moves via diffusion and convection [5]. Contact-independent interactions can be local (mainly between neighboring cells) or global (within the whole population), depending on the profile of the concentration gradient. *Saccharomyces cerevisiae* for instance uses its extracellular enzyme invertase to split sucrose, resulting in a glucose and fructose gradient around the cell. At high sucrose concentrations both aggregated and single yeast cells can grow (global interactions), while at low sucrose concentrations only aggregated yeast cells grow (local interactions) [8]. A similar pattern is observed for the extracellular protease of *Lactococcus lactis*, which activity results in a peptide gradient around the cell. At high cell densities both protease positive and protease negative cells grow, while at low cell densities mainly protease positive cells grow, since only they can benefit from their produced peptides [9].

Whether contact-independent interactions are local or global depends on the distance between cells and the concentration gradient profile, which is affected by the metabolite source, the metabolite-sink and the diffusion and convection rate between them. The metabolite source can for instance be a producer cell [10], a nutrient pool in the environment [11] or an extracellular enzyme [8, 9]. The metabolite-sink can be a metabolite consuming cell [12, 13], a metabolite degrading enzyme [14], liquid flow [15], or the volume of the system, as dilution reduces the metabolite concentration [8, 9]. Although the exact nature of the source and sink are often only implicitly mentioned in these studies, their importance is well-known. Costly cooperative interactions are for instance more likely to evolve when cells are close to each other, because cooperators compete with wild-type noncooperators for the excreted metabolite [5, 16, 17]. Selection for interactions is therefore often done by co-culturing cells on agar plates [18–20], and it is also described that interacting cells evolved aggregating phenotypes [21].

These examples show that in the presence of a metabolite-removing sink, concentration gradients constrain the distances over which interactions can occur. It is however not clear at what distances such interactions occur. Previous studies either quantified these distances in two-dimensional systems [11–13] or without a metabolite-removing sink [13, 14], while natural microbial communities reside in three-dimensional environments in which competing metabolite consumers and other types of metabolite-removing sinks are very likely to be present. We therefore combined computational and experimental analyses to provide a more systematic and quantitative perspective on the impact of cell-to-cell distance on metabolic interactions in three dimensions and in the presence of metabolite-removing sinks. We focused on the diffusion of glucose in a static, aqueous system. The reaction-diffusion model and experimental results show that in these conditions receiver cells cannot interact with glucose producing cells that are fixed at an average distance of 15 µm. However, producer–receiver aggregation facilitates metabolic interactions even when receiver cells have a low affinity for the product, caused by genetic variation of their glucose import systems. These results suggest that for sugars, organic acids, and amino acids competition or other metabolite-removal in a three-dimensional aqueous system reduces metabolic interaction distances to the low µm-range.

## Materials and methods

### Strains and media

All the strains that were used are listed in Table 1. *L. lactis* NZ9000 strains PTSman_GFP, PTScel_GFP, and GlcU_GFP were obtained by a single-crossover integration of vector pSEUDO::*Pusp45-gfp* [22] into the *pseudo* 10 locus on the chromosome of *L. lactis* NZ9000∆*ptcC*∆*GlcU*, NZ9000∆*ptnABCD*∆*GlcU*, and NZ9000∆*ptnABCD*∆*ptcC* [23], respectively. Integration was performed as previously described [24]. Transformants were selected on M17-agar plates supplemented with glucose, sucrose, and 5 µg/mL erythromycin. In all GFP-positive strains GFP-expression was under control of the constitutive *usp45* promotor.

**Table 1:** Bacterial strains and plasmids used in this study.

*L. lactis* strain	Description	Reference
NZ9000*∆ptcC∆GlcU*	Derivative of *L. lactis* NZ9000 containing a 1254 bp deletion in *ptcC* and a 864 bp deletion in *GlcU*.	[23]
NZ9000*∆ptnABCD∆GlcU*	Derivative of *L. lactis* NZ9000 containing a 1736 bp deletion in *ptnABCD* and a 864 bp deletion in *GlcU*.	[23]
NZ9000*∆ptnABCD∆ptcC*	Derivative of *L. lactis* NZ9000 containing a 1736 bp deletion in *ptnABCD* and a 1254 bp deletion in *ptcC*.	[23]
MG5267	*L. lactis* MG1363 with the lactose operon integrated into the genome.	[27]
NZ9000 Glc-Lac+	NZ9000∆*glk*∆*ptnABCD* containing a 657-bp deletion in *ptcBA*, carrying pMG8020 (lactose mini-plasmid of 23.7 kb, containing *lacFEGABCD*, derivative of pLP712).	[28]
MG1363_GFP	*L. lactis* MG1363 carrying pSEUDO::*P*_*usp45*_*-gfp*.	[22]
NZ9000_PTSman_GFP	Ery^r^, NZ9000*∆ptcC∆GlcU* carrying pSEUDO::*P*_*usp45*_*-gfp*.	This work
NZ9000_PTScel_GFP	Ery^r^, NZ9000*∆ptnABCD∆GlcU* carrying pSEUDO::*P*_*usp45*_*-gfp*.	This work
NZ9000_GlcU_GFP	Ery^r^, NZ9000*∆ptnABCD∆ptcC* carrying pSEUDO::*P*_*usp45*_*-gfp*.	This work

Plasmids	Description	Reference
pSEUDO::*P*_*usp45*_*-gfp*	Ery^r^, integration vector, pSEUDO::*P*_*usp45*_*-sfgfp(Bs)* derivative, carrying the gene coding for the green fluorescent protein (Dasher-GFP).	[22]

*L. lactis* was grown in chemically defined medium (CDM) described by Otto et al. [25], with the following changes: 0.6 g/L NH_4_-citrate, 2.5 mg/L biotin, 0.02 mg/L riboflavin, and no folic acid. *L. lactis* NZ9000 Glc-Lac+ was pre-cultured in CDM + 0.95 wt% lactose, *L. lactis* MG5267 in CDM + 0.5 wt% lactose and *L. lactis* MG1363 [26], *L. lactis* MG1363_GFP, *L. lactis* NZ9000_GFP_PTSman, *L. lactis* NZ9000_GFP_GlcU and *L. lactis* NZ9000_GFP_PTScel in CDM + 0.5 wt% glucose. Agarose beads contained CDM + 0.4 wt% carbon source and were incubated surrounded by oil, or by CDM + 0.2 wt% carbon source. Mono-cultures were incubated with the same carbon source as their pre-culture, co-cultures were incubated in the presence of lactose. Incubations were done at 30 °C for at least 16 h.

### Aggregation protocol

Producer and receiver cell pre-cultures (10 mL) were washed three times with 0.9% sodium chloride. Receiver cells were resuspended in 2 mL 0.225% sodium chloride, producer cells were resuspended in 0.9% sodium chloride and diluted to an OD_600_ of 1.1. Both were incubated in an ultrasonification bath (Branson 200 Ultrasonic cleaner, Branson Ultrasonics, Danbury, CT, USA) at 46 kHz for 3 min to ensure complete resuspension to single cells.

The surface of (non-)producer cells was charged positively by electrostatic deposition of polyethyleneimine (PEI; Mr 600,000–1,000,000; ~50% in H_2_O; Sigma-Aldrich, Saint Louis, MO, USA) as follows. Sonicated producer cells were mixed with 0.25% PEI (hydrated, pH 7) in a 1:1 (v/v) ratio and incubated at the room temperature for 5 min. After incubation cells were collected by centrifugation (900 *g*, 3 min) and washed by replacing the supernatant with 0.9% sodium chloride five times without resuspending the pellet. Washed cells were resuspended in 200 µL 0.9% sodium chloride and sonicated as described above. The surface of washed receiver cells was negatively charged and therefore not further modified [27]. Cell concentrations in the prepared producer and receiver suspensions were measured with flow cytometry (Accuri C6, BD Biosciences, San Jose, CA, USA).

Aggregates were formed electrostatically by mixing the positively charged producer cells with the negatively charged receiver cells, such that the oppositely charged cells stuck to each other. The suspension with negatively charged receiver cells was mixed using a T10 basic ULTRA TURRAX homogenizer with an S10N-5G dispersing element (IKA, Staufen, Germany) at 8000 rpm for 15–20 min. While mixing, the positively charged producer cells were added to the negatively charged receiver cells using a 1 mL syringe (Terumo, Tokyo, Japan), a Chemyx Fusion 200 syringe pump (125–400 µL/h, Chemyx Inc., Stafford, TX, USA) and polyethylene tubing (inner diameter 0.38 mm, BD, Franklin Lakes, NJ, USA). The mixing time (15–20 min) and syringe pump flow rate (125–400 µL/h) were adjusted within the mentioned ranges such that the final aggregate percentage was ~3%.

### Agarose beads formation and analysis

Agarose beads in oil were made by mixing a water and an oil phase. The oil phase contained Novec HFE 7500 fluorinated oil (3 M, Maplewood, MN, USA) and 0.2% PicoSurf 1 surfactant (Sphere Fluidics, Cambridge, UK). The water phase contained CDM, 1 wt% melted agarose with ultra-low gelling temperature (Type IX-A, A2576, Sigma-Aldrich, Saint Louis, MO, USA) and cells, and it was prepared as follows. Pre-cultures were washed with phosphate-buffered saline and the OD_600_ was measured to determine the cell concentration (assuming OD 1 = 10^9^ cells/mL). The total cell concentration in the aggregate suspension was determined using flow cytometry (Accuri C6). The producer cell or aggregate concentration in CDM with agarose was set to 2.7 × 10^6^/mL, the receiver cell concentration to 8.9 × 10^7^ cells/mL.

300 µL water phase and 700 µL oil phase were mixed using a T10 basic ULTRA TURRAX homogenizer with an S10N-5G dispersing element at 8000 rpm for 5 min, resulting in the formation of water-in-oil emulsions. The agarose in the water phase was subsequently solidified by placing emulsions on ice for at least 20 min, yielding agarose beads surrounded by oil. After solidification cells were fixed in the agarose matrix and growth therefore resulted in micro-colony formation within the agarose bead. Formed agarose beads had an average volume of 26 pL (diameter of 37 µm, Supplementary information section 1, Figs. S1 and S2). The distribution of cells over droplets in such an emulsion is described by a Poisson distribution [28]. This means that with an average volume of 26 pL and the cell concentrations described above, each bead contained on average 8 receiver cells. In addition to the receivers, ~2% of the beads contained two or more producer cells/aggregates and ~19% contained one producer cell/aggregate (~79% contained no producer/aggregate). The cell concentration in a 26 pL bead containing eight receiver cells and one producer is 3 × 10^8^ cells/mL. Assuming a homogenous spread of cells within agarose beads results in an average distance of 15 µm between cells. Microscopy images of agarose beads with cells confirmed this homogenous spread, and indicate that the average distance between cells was indeed close to 15 µm (Fig. S2).

To incubate agarose beads in CDM, 1 mL CDM and 1 mL perfluorooctanol (PFO, Alfa Aesar, Ward Hill, MA, USA) were added to the emulsion after solidification. This leads to the breaking of the emulsion and separation of the water and oil phase upon gently mixing. Subsequently the water phase, containing agarose beads in CDM, was separated from the oil phase and incubated for at least 16 h, while rotating. For incubation in the presence of competing glucose-consumers 10^9^ *L. lactis* MG1363 cells per mL were added to the CDM surrounding the agarose beads (Supplementary information section 2, Fig. S3).

Growth in agarose beads was analyzed with flow cytometry (Accuri C6). Agarose beads in CDM were measured directly. For agarose beads in oil the emulsion was first broken by adding 240 µL PBS and 300 µL PFO to 60 µL emulsion, followed by gently mixing. The water phase, containing agarose beads in PBS, was separated from the oil phase and measured using flow cytometry. Details about the flow cytometry gating strategy and data analysis are shown in Supplementary information section 3, Figs. S4 and S5.

### Reaction-diffusion models

Numerical reaction-diffusion models were implemented in COMSOL Multiphysics (COMSOL 5.0, Comsol Inc., Burlington, MA, USA).

For simulations in two- and three-dimensional systems without receiver cells, a glucose producing cell with a diameter of 1 µm was placed in the middle of a very thin rectangular block (1.1 µm thickness) to represent a quasi-two-dimensional system, or in the middle of a cube with the same volume to a represent three-dimensional system. For the cube, the concentration at the cube boundaries were set to zero (metabolite-sink) or insulated (no-flux boundary condition, no metabolite-sink). For the thin plate the concentration at the four lateral faces were set to zero, and the top and bottom boundaries were set to zero (metabolite-sink) or insulated (no-flux boundary condition, no metabolite-sink). The total volume of both systems was 1 nL (1 × 10^6^ cells/mL). The diffusion coefficient of glucose in water (D_s_) was based on literature values and set to 6.7 × 10^−10^ m^2^/s (aqueous conditions), to 25% of D_s_ (biofilm) or to 10% of D_s_ (colony) [29, 30].

For simulations in three-dimensional systems with agarose beads and producer and receiver cells, two spherical agarose beads were placed in a cubic computational domain. One bead contained a producer cell that secreted glucose with a constant rate, and both beads contained eight receiver cells that consumed glucose based on Monod (saturation) kinetics. The concentration at the agarose bead surface resulted from a partition coefficient which was set to 0 to model incubation in oil, and to 1 to model incubation in CDM. The diffusion coefficient of glucose (D_s_) was the same inside and outside agarose beads [31], and 10 times lower in microcolonies [29, 30].

Time-dependent studies of these systems yielded the spatial distribution of glucose. Supplementary information section 4 gives more details about the geometry (Fig. S6), used parameters (Table S1) and simulation results (Figs. S7–S11, Table S2).

## Results

### Reaction-diffusion modeling predicts short metabolic interaction distances in three-dimensional systems with a metabolite-sink

**Fig. 1:**
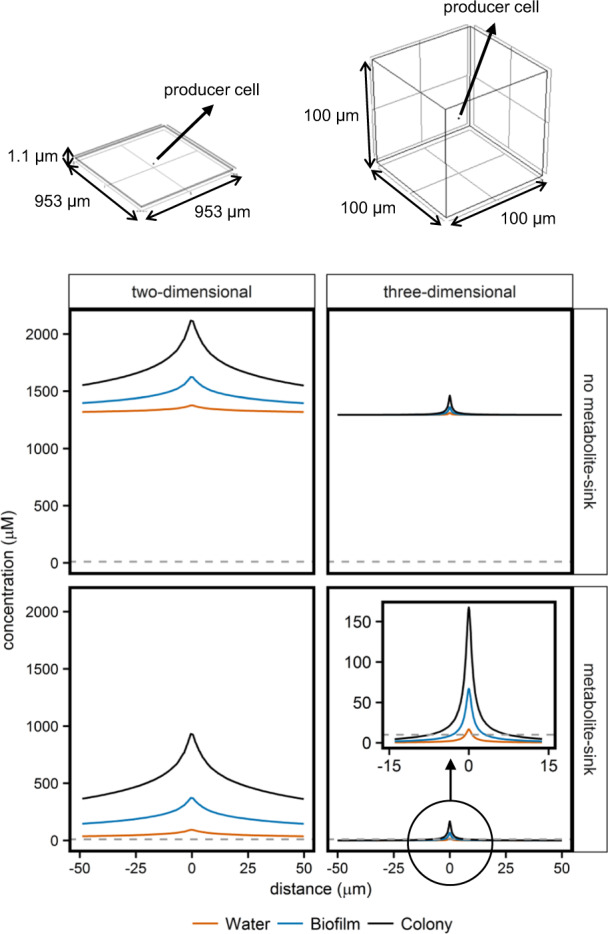
Predicted concentration gradients in two- and three-dimensional reaction-diffusion systems. Glucose producing cells were placed in a two- or a three-dimensional space, in the presence and absence of a metabolite-sink. Different environments were simulated by altering the diffusion coefficient. The diffusion coefficient of glucose in water (D_s_) was set to 6.7 × 10^−10^ [30], the diffusion coefficient of glucose in a biofilm (D_eff,biofilm,s_) was set to 0.25 times D_s_ [30], and the diffusion coefficient of glucose in a colony (D_eff,colony,s_) was set to 0.10 times D_s_ [29, 30] (Table S2). A time-dependent study in COMSOL Multiphysics yielded concentration gradients at several moments. The figure shows the concentration over a horizontal line crossing the producer cell, after 5 h of incubation. To aid visibility the *x*-axis-range was made similar for both plots, so for the two-dimensional system only part of the concentration gradient profile is shown. The dashed horizontal line indicates a concentration of 10 µM.

The basic ideas behind diffusion and the resulting concentration gradients are well-understood. To better understand the biological impact of these concentration gradients, we made reaction-diffusion models where concentration gradient profiles around a producer cell were calculated either in a cube to mimic a three-dimensional system, or in a thin plate to mimic a two-dimensional system (plate thickness of 1.1 µm, roughly matching the producer cell diameter of 1 µm, Fig. 1). In both cases the total volume was 1 nL (10^6^ cells/mL). We used the diffusion coefficient of glucose as it is similar to that of other sugars, organic acids, and amino acids [30], compounds relevant in many metabolic interactions. Factors like viscosity, the presence of extracellular polymeric substances and the local cell concentration vary between environmental conditions, and affect the diffusion coefficient [30, 32]. To visualize their effect on concentration gradient profiles we modeled diffusion in conditions representing aqueous, biofilm, and colony environments (Table S2).

In simulations without a metabolite-sink the produced glucose accumulated (Fig. 1). After 5 h the minimal glucose concentration was around 1500 µM in both two- and three-dimensional systems (Fig. 1, Table S2), indicating that glucose is biologically available (i.e. above the threshold for growth based on transporter affinities) in the whole system. The introduction of a metabolite-sink limited the glucose accumulation, resulting in concentrations close to 0 µM. In the aqueous two-dimensional system the glucose concentration dropped below a threshold of 10 µM (approximate threshold for growth based on transporter affinities) at a distance of 269 µm from the producer, while in a three-dimensional system this threshold was already reached at 0.7 µm. A decrease in the diffusion coefficient increased the distance at which the glucose concentration dropped below 10 µM, but predicted distances were still in the low µm-range (<7.5 µm, Fig. 1, Table S2).

Together these results indicate that in three-dimensional systems with a metabolite-sink metabolic interaction distances might be reduced to around the size of single cells.

### Design of a synthetic consortium and three-dimensional spatial structure for growth

**Fig. 2:**
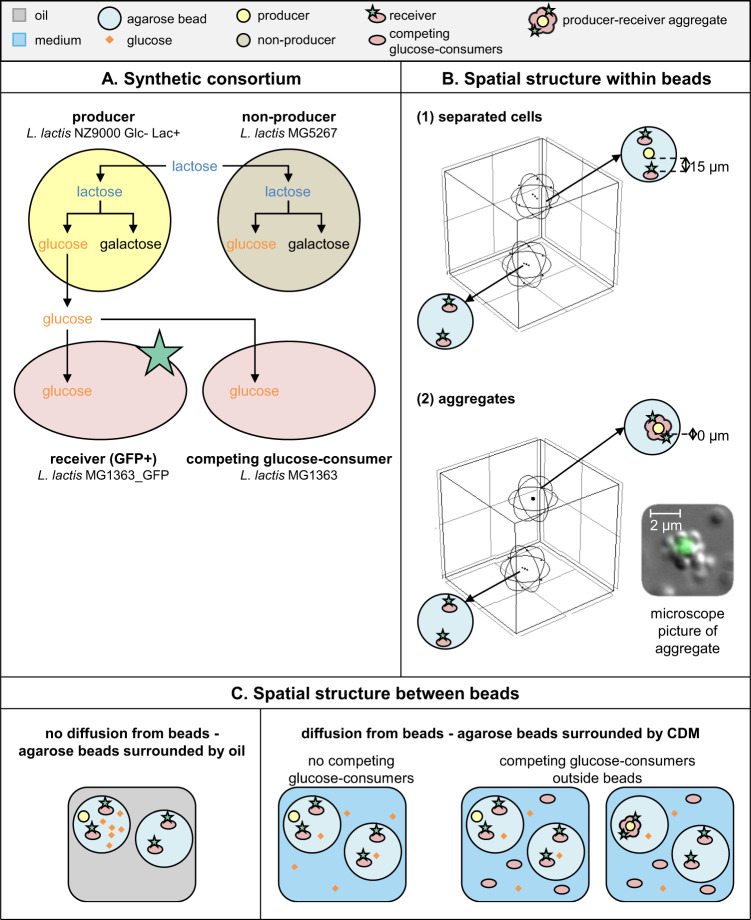
Metabolic interactions in three-dimensional spatially structured environments. **A** The four *L. lactis* strains that were used to make synthetic consortia: (1) “producers” which take up lactose and secrete glucose, (2) “receivers” which take up glucose and express GFP, (3) “non-producers” which take up lactose but do not secrete glucose and (4) “competing glucose-consumers” which take up glucose (Supplementary information section 2, Fig. S3). **B** The three-dimensional spatial structure within agarose beads. A distance of 15 µm between cells is comparable to a homogeneous distribution of 3 × 10^8^ bacteria/mL (Fig. S2). The microscopy image shows an aggregate where for visibility reasons a GFP-expressing cell is surrounded by nonfluorescent cells. Aggregates used in experiments were formed oppositely: a (non-)producer cell was surrounded by GFP-expressing receivers. **C** The three-dimensional spatial structure between agarose beads. Aggregates were only incubated in the presence of competing glucose-consumers.

To predict how glucose concentration gradients constrain interactions between micro-organisms in a three-dimensional environment, we extended the cubic model to contain producer and receiver cells (Supplementary information section 4.1 and 4.2, Fig. S6 and Table S1), and analyzed the impact of cell-to-cell distance on the interaction. To experimentally validate the model results we constructed synthetic consortia using four *L. lactis* strains. (1) A “producer” that takes up lactose and hydrolyzes it intracellularly to glucose and galactose. It was engineered to not metabolize glucose, which was therefore secreted while the cells grew on galactose. (2) A GFP-expressing “receiver” that can take up and grow on glucose, but not lactose. As receivers can only grow on glucose, their growth is indicative for the glucose availability at their position. (3) A “non-producer” that takes up lactose. It uses both the glucose and galactose moiety for growth, and therefore does not secrete glucose. (4) A “competing glucose-consumer” (Fig. 2A). To co-culture these cells in a three-dimensional system, glucose-producers and -receivers (the unidirectional cross-feeders) were encapsulated in solidified agarose beads with an average diameter of 40 µm. *L. lactis* was chosen because compared to other model organisms (e.g. *Escherichia* *coli*, *S. cerevisiae*) its metabolism and biomass yield are not sensitive to variations in oxygen and it can reach high cell concentrations inside these agarose beads. For negative controls glucose-producers were replaced by glucose-“non-producers”. Cells were embedded in the beads either as separate cells (on average 15 µm between cells, Supplementary information section 1, Fig. S2) or as aggregates (0 µm between cells, Fig. 2B). During incubation agarose beads were separated either by oil or by CDM (Fig. 2C). Separation by oil prevented diffusion of glucose from beads and therefore each agarose bead acted as an individual compartment. This enabled us to validate if cells could grow and interact in agarose beads. Separation by CDM resulted in glucose diffusion from beads, enabling us to study unidirectional cross-feeding in the presence of a concentration gradient in a three-dimensional system. To investigate the effect of metabolite-removal on the interaction distances, interactions were analyzed in the presence and absence of competing glucose-consumers outside the beads (Fig. 2C).

### Producer and receiver cells can grow and interact within agarose beads

**Fig. 3:**
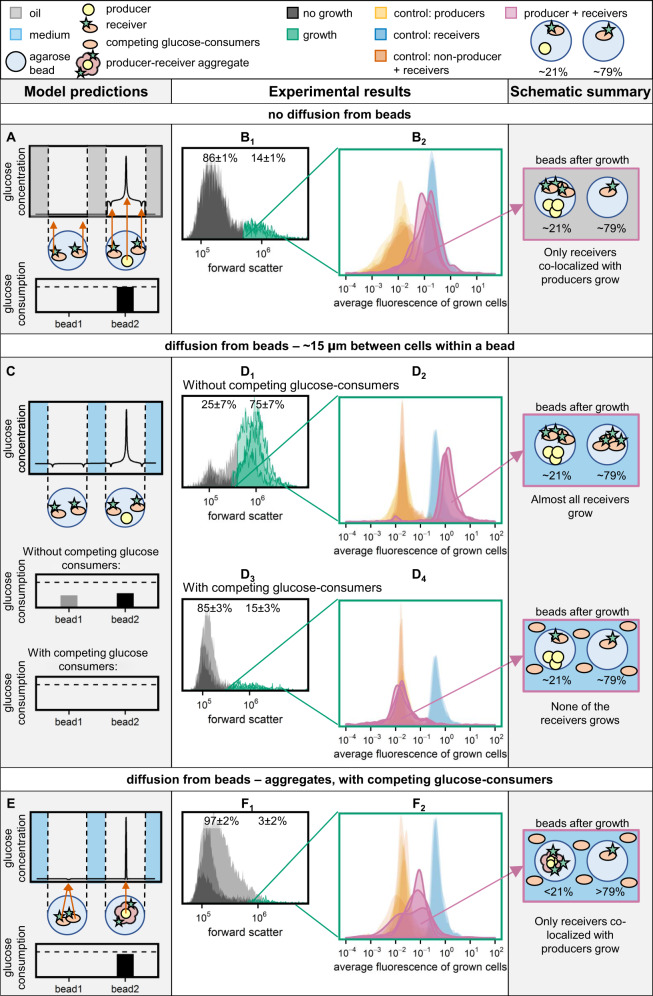
Consortium response in different spatial structures. **A**, **C**, and **E** show the predicted concentration gradient at the diagonal of the cube, for the following spatial structure: **A** no diffusion from beads, **C** diffusion from beads, 15 µm between cells within a bead, and **E** diffusion from beads, aggregated cells within a bead. For each condition the glucose uptake (mol/s) after 5 h of incubation was calculated, without considering growth of the cells. Bar plots show the glucose consumption rate (mol/s) in bead 1 (bead without producer cell) and bead 2 (bead with producer cell). Dashed lines indicate the glucose production rate (mol/s) of producer cells, which is equal in all conditions. Produced glucose that is not consumed in bead 1 or bead 2 is consumed by the competing glucose-consumers. In **B**, **D**, and **F** the experimental results are shown for these different spatial structures. Details about the gating strategy are given in Supplementary information section 3, Figs. S4 and S5. Density plots B_1_, D_1_, D_3_, and F_1_ show the populations that were gated as “growth” in the producer–receiver co-cultures (*n* = 3, around 3000 agarose beads measured per replicate). Density plots B_2_, D_2_, D_4_, and F_2_ show the average fluorescence of the grown cells (*n* = 3). This average fluorescence scales with the receiver cell fraction, as shown by the control samples that are added in the same plot: receivers only, producers only, and co-cultures of non-producers and receivers (*n* = 3 for each of them). The non-producers and receivers and the producers only controls are overlapping in all plots. The schematic drawing at the right summarizes the results from the presented density plots.

To analyze if we could detect growth in agarose beads, we cultured producers and receivers in beads surrounded by medium with glucose or lactose. We analyzed the beads before and after incubation using flow cytometry. When cells were incubated in the presence of a carbon source they could not use, the forward scatter before and after incubation was similar (Fig. S5). However, after incubation in the presence of a carbon source on which cells could grow the forward scatter was increased significantly, and we could separate beads with and beads without growth from each other using a forward scatter threshold (Figs. S4 and S5). To subsequently analyze if only producer cells, only receiver cells or both were grown, we calculated the average fluorescence of the grown cells (Supplementary information section 3, Fig. S4). As the population of grown cells can consist of only producer cells, only fluorescent receiver cells, or both, we expect the average fluorescence of the grown cells to scale with the receiver cell fraction within the population of grown cells. Consistent with this expectation the average fluorescence of the grown cells was low for beads with producers and high for beads with GFP-expressing receivers (Fig. 3B_2_). Together these results show that using the forward scatter signal we can detect in which percentage of the beads cells could grow, and using the average fluorescence of the grown cells we can detect which cell-types were grown within these beads.

To compare growth in agarose beads with growth in liquid medium, we estimated the number of receivers in a fully grown agarose bead by dividing the total fluorescence of an agarose bead by the fluorescence of single receiver cells. These measurements indicated a cell concentration of 7.8 × 10^8^ ± 0.7 × 10^8^ cells/mL in fully grown agarose beads, which is similar to what is reached in liquid medium with the same glucose concentration (7.4 × 10^8^ cells/mL). These results show that the compartmentalization method and the incubation conditions do not affect the biomass yield of receiver cells.

To validate that cells could not only grow, but also interact within agarose beads, we made beads with producer and receiver cells. Producer cells could always grow, while receivers could only grow when glucose secreted by producers was available to them (Fig. 2A). Based on the chosen droplet loading all beads contained receiver cells and ~21% of them also contained a producer cell. The results show that after incubation 14 ± 1% of the agarose beads showed an increased forward scatter, indicating growth (Fig. 3B_1_, Table 2). As this is close to 21%, this indicates that there was only growth in beads containing producers and receivers. Within these beads the grown cells had a high average fluorescence (Fig. 3B_2_), indicating that receiver cells grew on glucose provided by producer cells. Negative controls with non-producers instead of producer cells showed growth of non-producer cells only (low average fluorescence of the grown cells, Fig. 3B_2_, Table 2), confirming cross-feeding between producer and receiver cells.

**Table 2:** Consortium response in different spatial structures.

Incubation condition	Average cell-to-cell distance	Producer + receivers (~21%), receivers only (~79%)		Non-producer + receivers (~21%), receivers only (~79%)	
		Beads with growth	Average fluorescence of grown cells	Beads with growth	Average fluorescence of grown cells
No diffusion from beads	15 µm	14 ± 1%	80 ± 11%	16 ± 0%	17 ± 4%
Diffusion from beads, no competing glucose consumers	15 µm	75 ± 7%	131 ± 6%	6 ± 1%	1.3 ± 0.4%
Diffusion from beads, competing glucose consumers	15 µm	15 ± 3%	−3.5 ± 4.7%	9 ± 2%	−4.5 ± 2.7%
	0 µm	3 ± 2%	39 ± 7%	4 ± 2%	−0.7 ± 10%

Summary of the experimental results of Fig. 3. The columns “beads with growth” give the percentage of beads that were gated as “growth” (*n* = 3, mean ± sd) for co-cultures of producers and receivers (corresponding to the density plots in Fig. 3B_1_, D_1_, D_3_, F_1_), and for co-cultures of non-producers and receivers. The columns “average fluorescence of grown cells” give the fluorescence of grown cells on a scale of 0–100% (*n* = 3, mean ± sd). The median of the log(average fluorescence of grown cells) of the producer-control sample was set to 0% and the median of the log(average fluorescence of grown cells) of the receiver-control sample to 100%. A higher percentage indicates more growth of receiver cells. The average fluorescence of grown cells can exceed the value of the receiver-control sample (see for example Fig. 3D_2_), causing the percentage to be higher than 100%. Corresponding density plots are shown in Fig. 3B_2_, D_2_, D_4_, F_2_.

Altogether this setup forms a synthetic consortium where spatial interactions can be manipulated in a three-dimensional environment, and which allows the detection of growth and interactions using flow cytometry. It furthermore shows that when surrounded by oil, receiver cells only grow when localized in beads with producer cells.

### Under glucose competition receivers cannot interact with producers that are on average 15 µm away

In the example above glucose could not diffuse from beads and each agarose bead acted as an individual compartment. In contrast, when glucose can diffuse from agarose beads the model predicted that the glucose concentration flattens close to the producer. In that case receivers at a distance of 15 µm from a producer in the same bead are exposed to similar glucose concentrations as receivers in beads without a producer (Fig. 3C). When the producer and receiver cells are randomly distributed within the agarose beads, the glucose uptake of receivers might change depending on their positioning. However, in almost 60% of the simulated configurations the glucose uptake changed less than twofold compared to the default positioning from Fig. 2B, and in over 90% the change was less than fourfold (Fig. S9). To reduce the computational time, we used the default positioning in subsequent simulations.

If the model prediction that all receivers see similar glucose concentrations is correct, we expect that most receivers can grow when the global glucose concentration builds up (Fig. 1, Table S2). However, in case of glucose competition the glucose concentration is expected to stay low and even receivers 15 µm away from a producer in the same bead should not be able to grow. To test these predictions we incubated agarose beads in CDM, which allows glucose diffusion from beads. Without competing glucose-consumers in the medium outside the beads 75 ± 7% of the beads showed growth and the average fluorescence of the grown cells was high (Fig. 3D_1_, D_2_ and Table 2). The percentage of beads showing growth is higher than the percentage of beads containing a producer cell (around 21%), indicating growth of both receivers with and receivers without a producer in their bead. When we took the same beads but added competing glucose-consumers outside the beads, only 15 ± 3% of the beads showed growth. Within these beads the average fluorescence of the grown cells was low, indicating that only producers grew (Fig. 3D_3_, D_4_ and Table 2). These results are consistent with the model predictions (Figs. 3C and S9), and show that under glucose competition even microcolonies of producers cannot sustain growth of receivers that are on average 15 µm away.

Without competing glucose-consumers still 25 ± 7% of the beads were gated as “no growth”, although the model predicted that all receivers could grow (Fig. 3C, D_1_). These beads could be false negatives caused by our stringent gating strategy (Supplementary information section 3, Figs. S4 and S5), or by empty beads with single fluorescent cells attached to their outside.

For the beads gated as “growth”, we observed an increased average fluorescence compared to the single receiver controls (Fig. 3D_2_). It is known that fluorescence of individual cells increases with decreasing growth rate [33, 34], suggesting that in co-cultures the higher average fluorescence could be caused by glucose limited and therefore slower growth of the receivers in the beads.

Together the data show that competition for glucose in a three-dimensional environment prevents interactions of cells that are on average 15 µm apart, because the presence of competing public good-consumers leads to steep concentration gradients.

### Aggregated producers and receivers interact even under glucose competition

In the presence of steep concentration gradients microbial interactions might be facilitated by bringing producers and receivers into close proximity. Consistently, the model predicted that cell aggregation would allow receivers to grow under glucose competition (Fig. 3E). We developed a protocol to make producer–receiver aggregates. Defined aggregates were formed by adding positively charged producers to an excess of negatively charged receivers, ensuring that producers were directly surrounded by receivers. In this way we obtained a mixture of single receivers and aggregates of one producer with approximately eight receivers attached to its surface (Fig. 2B). We roughly estimated the aggregate concentration in the mixture based on the added amount of positively charged producer-cells. These aggregates were subsequently encapsulated in agarose beads following a Poisson distribution, with the aim to add an aggregate to at most 21% of the beads. The actual percentage of beads containing a viable aggregate is likely lower, as not all producers remain viable, and as some aggregates contain multiple producer cells due to clumping. However, underestimating the percentage of beads with a viable aggregate would not affect the results, as we only analyze agarose beads with growth after incubation (Supplementary information section 3, Figs. S4 and S5).

We incubated the formed agarose beads in CDM with competing glucose-consumers. After incubation we saw an increased scatter in 3 ± 2% of the beads (Fig. 3F_1_, Table 2), indicating only growth in beads with both producers and receivers. Within these beads the average fluorescence of the grown cells was increased compared to the producer mono-culture (Fig. 3F_2_), indicating growth of both producers and receivers. Negative controls with non-producers and receivers showed an average fluorescence similar to the producer mono-culture, indicating growth of producer cells only (Fig. 3F_2_). We did not include samples without competing glucose-consumers, as Fig. 2 shows that all receivers will grow in these conditions.

Overall, the results show that close proximity through cell aggregation facilitates microbial interactions, even in a three-dimensional system with competition for the public good.

### Aggregation results in dense microcolonies, facilitating growth of receivers with low affinity and low *V*_max_ glucose transporters

Glucose uptake can be affected by cellular properties and environmental conditions. Consistent with Fig. 1, the model predicts that decreasing the diffusion coefficient has limited effect on the glucose uptake of receiver cells that are either 0 or 15 µm away from producer cells (Fig. S11). The model furthermore predicts that increasing the glucose production rate increases the interaction distance, but interactions are still limited to the low µm-range (Fig. S9). As in the presence of a glucose-sink the glucose concentration is low, we expected that the glucose affinity (*K*_m_) and maximal glucose uptake rate (*V*_max_) of receiver cells would affect the efficiency of interactions. This effect could however be counteracted by receiver-independent growth of producers, which increases the glucose production rate and therefore the local glucose concentration (Fig. S9), resulting in a similar glucose uptake of the different mutant-receivers. To study the effect of changes in *q*_s_^max^ in more detail, we modeled producer–receiver aggregates with receivers that contained one of the three different glucose transporter types of *L. lactis*—PTSman, PTScel, and GlcU, which are characterized in detail by Castro et al. [23] (Supplementary information section 4.5). Within aggregates the effective diffusion coefficient (D_eff,s_) is described to be 10–70% of the diffusion coefficient in water (D_s_), depending on the aggregates’ density [29, 30]. When we assume the presence of 50 producer cells and set D_eff,s_ to 10% of D_s_, the model predicts that receivers with the low *K*_m_ and high *V*_max_ transporter PTSman (*K*_m_ = 0.013 mM, *V*_max_ = 0.22 µmol/min/mg protein [23]) consume about 15 times more glucose than receivers with a high *K*_m_ or low *V*_max_ transporter (PTScel: *K*_m_ = 8.7 mM, *V*_max_ = 0.25 µmol/min/mg protein, GlcU: *K*_m_ = 2.4 mM, *V*_max_ = 0.08 µmol/min/mg protein [23]) (Supplementary information section 4.5, Fig. S10). When D_eff,s_ is 70% of D_s_, this difference is around 70-fold.

**Fig. 4:**
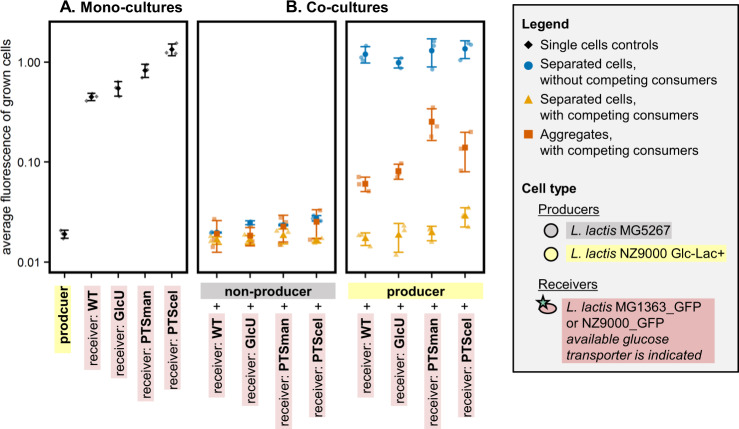
Effect of glucose transporter affinity and *V*_max_ on the receiver response. Mono- and co-cultures of (non-)producers and receivers with different glucose transporter types were incubated in agarose beads surrounded by CDM. Receivers are ordered based on their growth rate (Supplementary information section 5, Table S3). *L. lactis* NZ9000_ PTSman has a *K*_m_ of 0.013 mM and a *V*_max_ of 0.22 µmol/min/mg protein, *L. lactis* NZ9000_ PTScel has a *K*_m_ of 8.7 mM and a *V*_max_ of 0.25 µmol/min/mg protein, and *L. lactis* NZ9000_ GlcU has a *K*_m_ of 2.4 mM and a *V*_max_ of 0.08 µmol/min/mg protein [23] (Supplementary information section 4.5). **A** Producer mono-cultures were incubated in the presence of lactose, receiver mono-cultures in the presence of glucose. In producer mono-cultures agarose beads contained separated cells. **B** Co-cultures were incubated in the presence of lactose. Agarose beads contained either separated cells (on average 15 µm between cells within a bead), or producer–receiver aggregates (0 µm between cells). The beads were incubated in CDM with and without competing glucose-consumers. For each culture the median and standard error of the average fluorescence of the grown cells is shown (*n* = 3). Corresponding density plots are shown in Supplementary information section 6, Fig. S12.

To validate the model results, we constitutively expressed GFP in engineered *L. lactis* strains which each contained only one of the three glucose transporters PTSman, PTScel, or GlcU [35]. We subsequently analyzed if their glucose uptake was high enough to interact with producers. The experimental results show that in mono-culture controls the average fluorescence of the grown cells decreased with an increasing growth rate (Fig. 4 and Supplementary information section 5, Table S3), an effect that we saw before. When producers and receivers were on average 15 µm away from each other, the mutant-receivers show similar behavior as the wild-type-receiver: in the absence of competing glucose-consumers receivers grew independently of their distance from a producer cell, while in the presence of competing glucose-consumers producers could not sustain growth of receivers (Figs. 4b and S12). Fig. 4 further shows that in producer–receiver aggregates even the low-affinity receivers could grow. These results are consistent with the model prediction that at a high glucose production rate *q*_p_ and with the formation of dense microcolonies with a low D_eff,s_ the glucose uptake of the different mutant-receivers is high (Fig. S10). Aggregates with receivers containing the low *K*_m_ and high *V*_max_ transporter PTSman showed the highest average fluorescence of the grown cells. This finding is consistent with the model predictions that receivers containing PTSman have the highest glucose uptake rate.

Altogether the data show that in a three-dimensional system with a metabolite consuming sink a steep concentration gradient is obtained, and cells that are on average 15 µm away from each other cannot interact through glucose cross-feeding. This physical constraint can be overcome by bringing cells together in the low µm-range, as achieved through cell aggregation—physical contact.

## Discussion

Contact-independent interactions can be local or global, depending on the profile of the concentration gradient. Previous studies quantified interaction distances either in monolayers of cells (two-dimensional system) or in absence of a competing metabolite-sink [13, 14]. While these studies give valuable insight, they have a limited resemblance to environmental conditions, which are typically three-dimensional and harbor competing organisms and other metabolite-removing sinks. A reaction-diffusion model predicted that in three-dimensional systems in the presence of a metabolite-removing sink, a concentration of 10 µM glucose was reached at a two orders of magnitude shorter distance than in two-dimensional systems, reducing the interaction distances to around the size of single cells (Fig. 1, Table S2). This was the case in conditions representing aqueous, as well as biofilm and colony systems. These results suggest that in more realistic environmental conditions interaction ranges might be much shorter than the in vitro two-dimensional systems predict. In this study we therefore analyzed the impact of cell-to-cell distance on unidirectional cross-feeding of glucose in a three-dimensional aqueous environment, in the presence and absence of a competing metabolite consumer as a public good-removing sink.

We found a global receiver-response in the absence of competing glucose-consumers, and a local receiver-response in the presence of competing glucose-consumers (Fig. 3). Diener et al. observed a similar pattern of local and global interactions during *S. cerevisiae* mating [14]. Haploid cells secrete a peptide, which is sensed and degraded by haploid cells of the opposite mating type. This results in a local high peptide concentration and local interactions: cells from opposite mating types initiate mating specifically in each other’s direction. However, incubation of mutants that could not degrade the peptide resulted in a global high peptide concentration, and independent of their location cells initiated mating in different directions.

For wild-type cells Diener et al. predicted that the maximum information content of the peptide distribution is similar for cells ~17 and ~2 µm away from each other [14], suggesting that yeast cells interact efficiently when they are 17 µm away from each other. Similar interaction distances (3.2–12.1 µm) were found by Dal Co et al. when they grew bidirectionally cross-feeding *E. coli* cells in a microfluidic chamber [13]. Our data however show that in a three-dimensional environment with a metabolite-sink the interaction distances are shorter, as producers and receivers that were on average 15 µm away from each other could not cross-feed (Fig. 3D_3_, D_4_). These results match the model prediction that in the presence of a metabolite-sink, interaction distances in three-dimensional systems are shorter than in two-dimensional systems. In the set-ups of Dalco et al. and Diener et al. the metabolite is only degraded/consumed by the receiver itself, so the metabolite concentration will decrease close to receiver cells. When receivers compete with metabolite-sinks, such as competing metabolite consumers, continuous liquid flow or a dilute system, the overall metabolite concentration will be lowered, resulting in shorter interaction distances. Consistent with this idea Koschwanez et al. showed that at low cell densities and low sucrose concentrations, where the volume acts as the main metabolite-sink, *S. cerevisiae* cannot grow even though invertase splits sucrose into glucose and fructose in the periplasmic space, so very close to the receiver cell [8].

During evolution of cooperation in which costly compounds are secreted, wild-type noncooperators form a competing public good-sink. Our results therefore indicate that for glucose-like compounds in an aqueous environment, cell-to-cell distances in the low µm-range are required to evolve costly cooperation. Aggregation also allows (evolution of) contact-dependent transfer mechanisms, like nanotubes or vesicle chains. To our knowledge *L. lactis* does not exchange cytosolic material using these contact-dependent transfer mechanisms and the model indicates that just diffusion can explain our experimental results. When cells aggregate or grow in biofilms the diffusion distance and diffusion rate will be reduced [32, 36], and this might increase the efficiency of interactions [16, 37]. Efforts to understand the micro-scale structure of aggregates and biofilms might therefore reveal important information about the underlying interactions in natural communities [36, 38]. We furthermore found that high and low-affinity receivers and receivers with a low *V*_max_ glucose transporter were all unable to grow when they were on average 15 µm away from producers, but all did grow when aggregated with producers (Fig. 4). This suggests that dense microcolonies with a low diffusion rate were formed (Supplementary information section 4.5, Fig. S10). Aggregating cells therefore seem to kill two birds with one stone: they decrease both the cell-to-cell distance and the diffusion rate, two factors which were previously reported to promote interaction [16, 37].

However, aggregation also slows down the diffusion of inhibiting metabolic end-products from the micro-colony and the diffusion of extracellular nutrients into the micro-colony [39, 40], and it therefore not always increases the interaction efficiency. Aggregation of the cross-feeding yogurt consortium (*Lactobacillus bulgaricus* and *Streptococcus thermophilus*) in 100–300 µm capsules reduced for instance their growth and acidification rates, and proteolysis was only faster in the first hour [41], indicating that in this case the aggregation costs did not outweigh the benefits.

When producer cells can form aggregates or microcolonies independently of receiver cells (e.g. in unidirectional cross-feeding), the local metabolite production rate and hence the local metabolite concentration increases (Fig. S9). This might facilitate interactions, as shown for invertase positive aggregates of yeast cells [8]. Our study shows that even producer microcolonies that were on average 15 µm away from receiver cells could not sustain receiver-growth, emphasizing the importance of close proximity for both uni- and bidirectional cross-feeding systems. Future research could focus on bidirectional cross-feeding in aggregates: as the mutual dependency reduces the metabolite production rate factors like receiver affinity might play a bigger role here. The described platform can be used to study both aerobic and anaerobic organisms [42], and could furthermore be used to study for example the effect of confinement size (determined by the agarose bead size) or metabolite-sink strength.

In the presence of a metabolite-sink, interactions involving molecules with diffusion coefficients similar to glucose (e.g. other sugars, organic acids, amino acids [30]) require cell-to-cell distances in the low µm-range. Consistently, many extracellular substrate-degrading enzymes are attached to the cell, which places the source (enzyme) close to the receiver (cell). Invertase is for instance located in the periplasmic space of *S. cerevisiae* [43], the protease of *L. lactis* is attached to the cell wall [9] and in both fungi and bacteria cellulosomes are also attached to the cell wall [44]. Hauert et al. argue that when a producer also benefits from its own product, which is the case for extracellular enzymes, spatially structured localization of cells is only advantageous when the enzyme production costs are high [45]. Attachment of extracellular enzymes to the cell wall therefore suggests that these enzymes are costly. This is consistent with Bachmann et al., who showed in *L. lactis* that protease negative strains outcompeted protease positive strains with a cell wall bound protease, unless they were more than 1 mm apart (cell density lower than 10^3^ cells/mL) [9].

Larger compounds like enzymes have a lower diffusion coefficient [29, 46], which is expected to increase the interaction distances in the presence of a metabolite-sink (Fig. 1, Table S2). However, also here concentration gradients shaped the evolution of molecular mechanisms involved in the interaction. Slow diffusion of large, aggregated resources like particulate iron (>0.4 µm) can for instance cause cellular iron uptake to become diffusion limited. It is therefore hypothesized that cells increase their iron uptake rate by secreting siderophores that bind to iron particles, extract iron ions, and subsequently form fast diffusing iron-siderophore complexes [47].

Controlled metabolite exchange is a critical feature of living cells [48], and forms the basis for extracellular metabolism of nutrients and interactions with other cells. This study points to constraints—and opportunities—that concentration gradients may impose on cellular interactions, how it shaped their evolution and their role in microbial consortia, and how researchers can use these principles to understand and steer these interactions.

## Supplementary information

Supplementary information
