# Supplementary material for: Microbial competition reduces metabolic interaction distances to the low µm-range
Source: ISME J. 2020 Oct 19;15(3):688–701. doi: 10.1038/s41396-020-00806-9 (PMC8027890; doi:10.1038/s41396-020-00806-9)
Supplement: Supplementary file 1 — Supplementary information [file 41396_2020_806_MOESM1_ESM.pdf]

Supplementary information for the manuscript entitled "Microbial competition reduces metabolic interaction distances to the low µm-range"

Rinke J. van Tatenhove-Pel[a], Tomaž Rijavec[b], Aleš Lapanje[b], Iris van Swam[a], Emile Zwering[a], Jhonatan A. Hernandez-Valdes[c], Oscar P. Kuipers[c], Cristian Picioreanu[d], Bas Teusink[a], Herwig Bachmann[a,e*]

[a] Systems Biology Lab, Amsterdam Institute for Molecules, Medicines and Systems, VU University Amsterdam, de Boelelaan 1108, 1081HV Amsterdam, The Netherlands

[b] Department of Environmental Sciences, Jožef Stefan Institute, Jamova cesta 39, 1000 Ljubljana, Slovenia

[c] Department of Molecular Genetics, Groningen Biomolecular Sciences and Biotechnology Institute, University of Groningen, Nijenborgh 7, 9747AG Groningen, The Netherlands

[d] Department of Biotechnology, Delft University of Technology, Van der Maasweg 9, 2629HZ, Delft, The Netherlands

[e] NIZO Food Research, Kernhemseweg 2, 6718ZB Ede, The Netherlands

* Address correspondence to Herwig Bachmann, h.bachmann@vu.nl.

Section 1: Agarose bead size- and volume-distributions

We prepared agarose beads surrounded by oil and made microscopy images (9 per emulsion, Figure S1A shows an example). Images were subsequently analyzed with ImageJ to identify the beads (Figure S1B), and to measure size- and volume distributions (Figure S1C and S1D). Small droplets were not always identified, but as they contain only little volume this only marginally affects the analysis. Beads on the edge of the image were excluded from the analysis. Formed emulsions were polydisperse but distributions of replicates were reproducible, with mean volume ± SEM of 26±2 pL (diameter of 37 μm).

Figure S2 shows microscopy images of beads filled with on average 8 cells per droplet (Figure S2A) and empty beads (Figure S2B). *L. lactis* cocci and diplococci were visible in beads with cells, but not in empty beads. Only cells in the focal plane are visible in the image, and they seem homogeneously spread within beads. The average distance between cells was 18±7 μm (Figure S2D, n=123), which is consistent with the expected cell-to-cell distance of around 15 μm. An example of an analyzed figure is shown in Figure S2C.

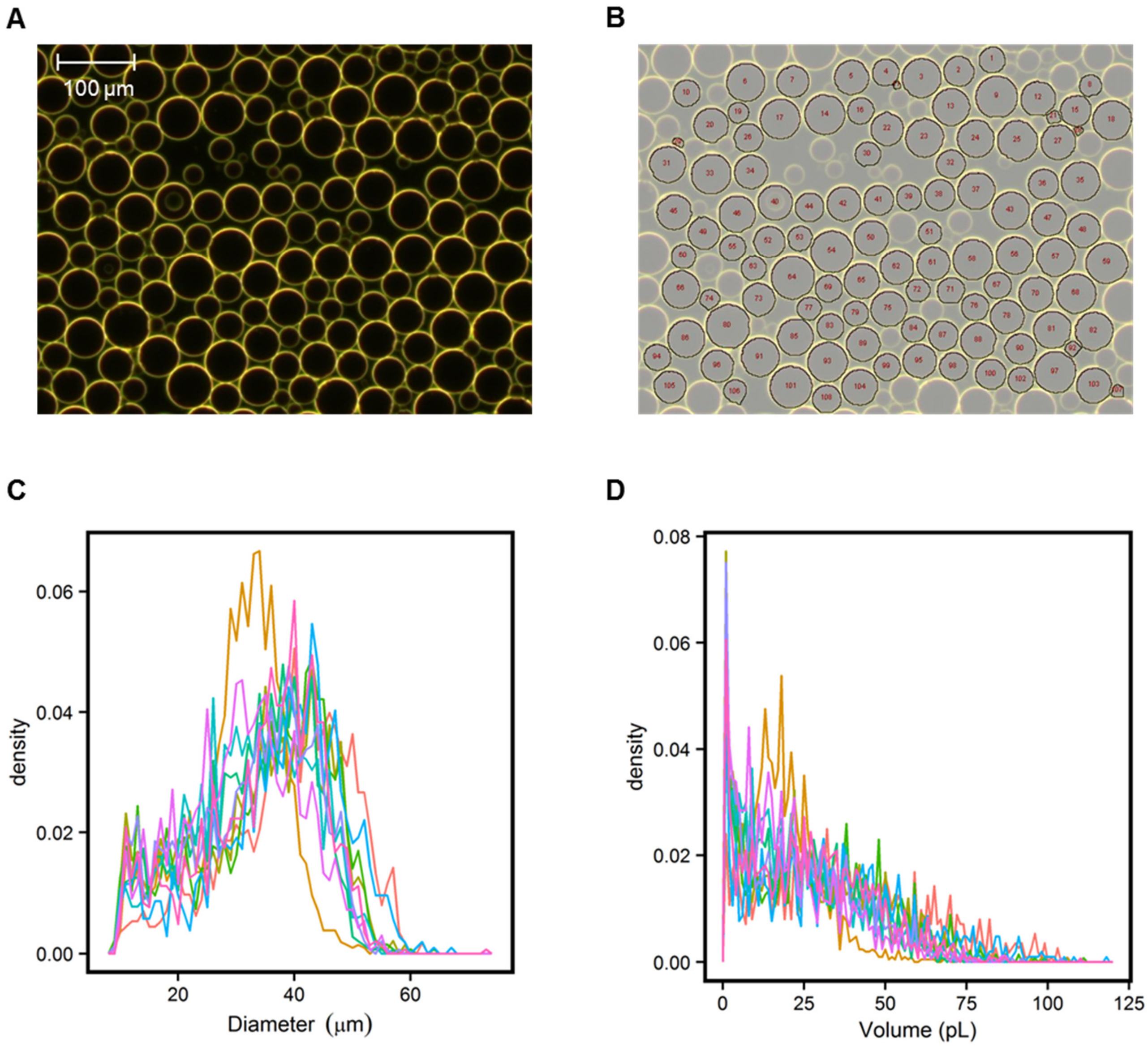

Figure S1. Agarose bead size and volume. (A) An example microscope image of agarose beads. **(B)** Agarose beads identified in (A) after ImageJ analysis. **(C and D)** Agarose bead size (C) and volume (D) distribution (n=10 emulsions, 9 images per emulsion).

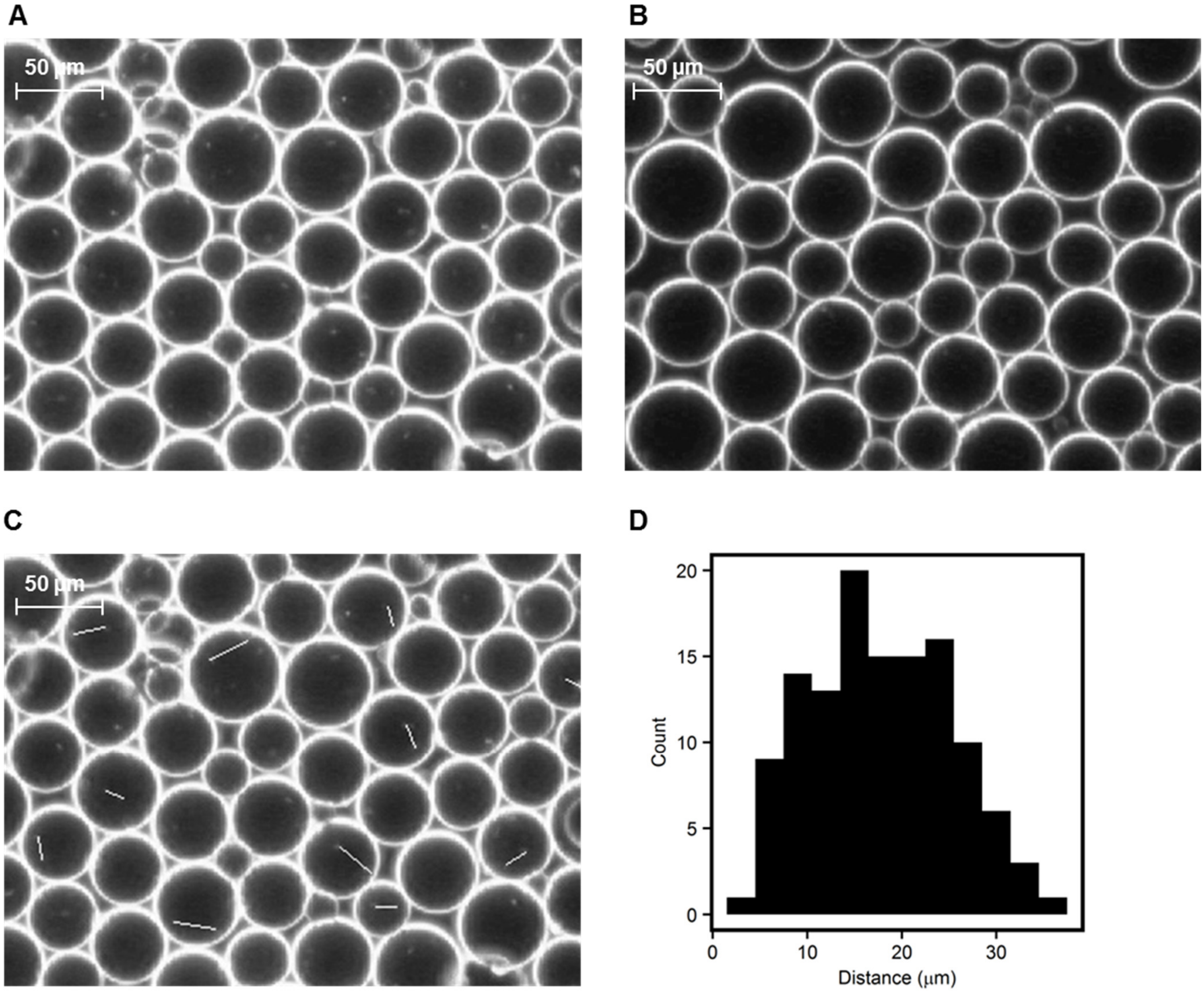

Figure S2. Cells in agarose beads. Microscopy images of agarose beads that contained on average 8 cells per bead (A) or agarose beads that were empty (B). For agarose beads that contained cells the distance between cells was measured (example in C), and plotted in a histogram (n=123) (D).

Section 2: 10^9 *L. lactis* MG1363 cells/mL as competing glucose-consumers

To establish if addition of 10^9 *L. lactis* MG1363 cells per mL as competing glucose-consumers outside agarose beads prevented cross-talk between beads, we mixed beads with producers and beads with receivers and incubated them in the presence of lactose in different spatial structures (Figure S3). After incubation surrounded by oil only producers were grown, which was expected as glucose could not diffuse from beads. When glucose could diffuse from beads and no *L. lactis* MG1363 cells were added outside the beads, both producers and receivers grew. However, in the presence of 10^9 *L. lactis* MG1363 cells per mL outside the agarose beads only producers grew, suggesting that glucose leaving beads with producers was mainly consumed by the competing metabolite-consumers outside the beads and did not reach receivers in neighboring beads. The glucose concentration outside the beads probably did not exceed the low micro-molar range, as the K_m for glucose of the highest affinity transporter in *L. lactis* MG1363 is 13 µM [1] and the *L. lactis* MG1363 cell density was high.

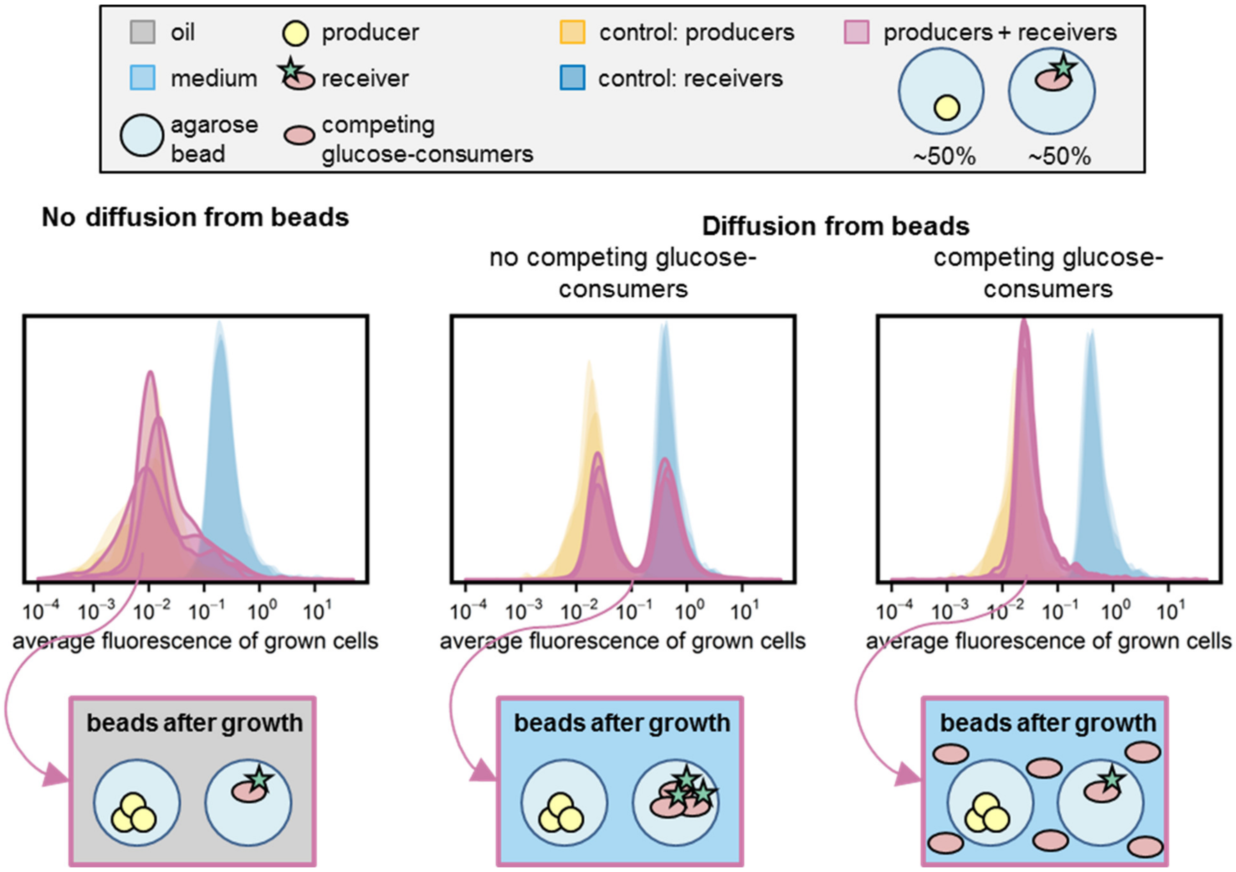

Figure S3: Addition of *L. lactis* MG1363 outside agarose beads prevents cross-talk between beads. Agarose beads with producers and agarose beads with receivers were mixed and incubated in three different spatial structures: surrounded by oil (no diffusion from beads, n=3), surrounded by CDM without *L. lactis* MG1363 (no competing glucose-consumers, n=3) and surrounded by CDM with 10^9 single *L. lactis* MG1363 cells per mL (competing glucose-consumers, n=3). Density plots show the average fluorescence of the grown cells. Schematics summarize the density plot results.

Section 3: Flow cytometry gating strategy and data analysis

Figure S4 shows the flow cytometry gating strategy and data analysis procedure.

Overview. The signals of single cells and agarose beads were identified using control samples with only agarose beads and only single cells. As their locations in the plot partially overlap, each sample with agarose beads containing cells was measured twice. In the first measurement we acquired an overview of the complete sample, containing both single cells and agarose beads. In the second measurements we used a forward and side scatter threshold to leave out most of the single cells. The resulting data contained mainly agarose beads, and was used for further gating and data analysis.

Step 1. Beads containing cells ("filled beads") were gated by including the largest agarose beads and excluding the noise and the empty agarose beads. For the control samples with only producers empty beads were not excluded, because before incubation agarose beads with non-fluorescent producers could not be separated from empty beads.

Step 2. Beads with and without growth were separated with a forward scatter threshold. To verify that the forward scatter of agarose beads only increases due to cell growth, we incubated beads with producers or receivers in the presence of glucose or lactose. Figure S5 shows that a significant increase in forward scatter was only observed when cells could grow. The forward scatter threshold was set for each sample individually, based on the forward scatter before incubation. This stringent gating ensures that beads without growth are excluded from analysis. This is important, as classifying beads without growth as "beads containing growth" would label receiver cells that were already present before incubation as "grown", which in turn would affect the average fluorescence of grown cells and therefore the interpretation of the data. The consequence of this stringent gating is that the amount of beads with growth might be underestimated, and the percentages of grown beads in Table 2 and Figure 3 should therefore be interpreted as minimum values.

Step 3. The side scatter and fluorescence were background-corrected using their values before incubation. Using these background-corrected values the fluorescence/scatter ratio of grown cells was calculated, which approximates the average fluorescence of the grown cells. The distribution of this average fluorescence of the grown cells was plotted to identify which cell-types were grown inside the agarose beads.

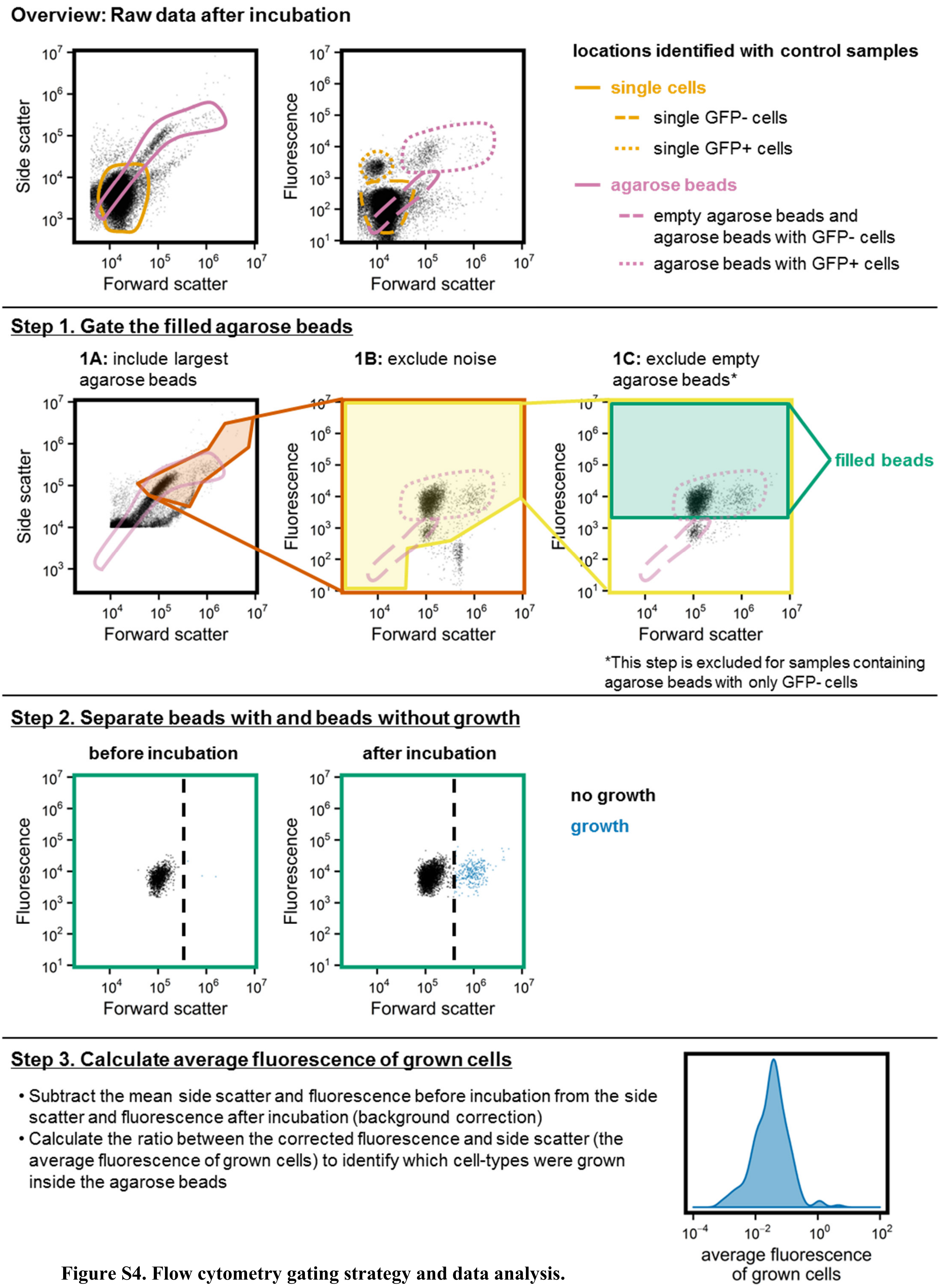

Figure S4. Flow cytometry gating strategy and data analysis.

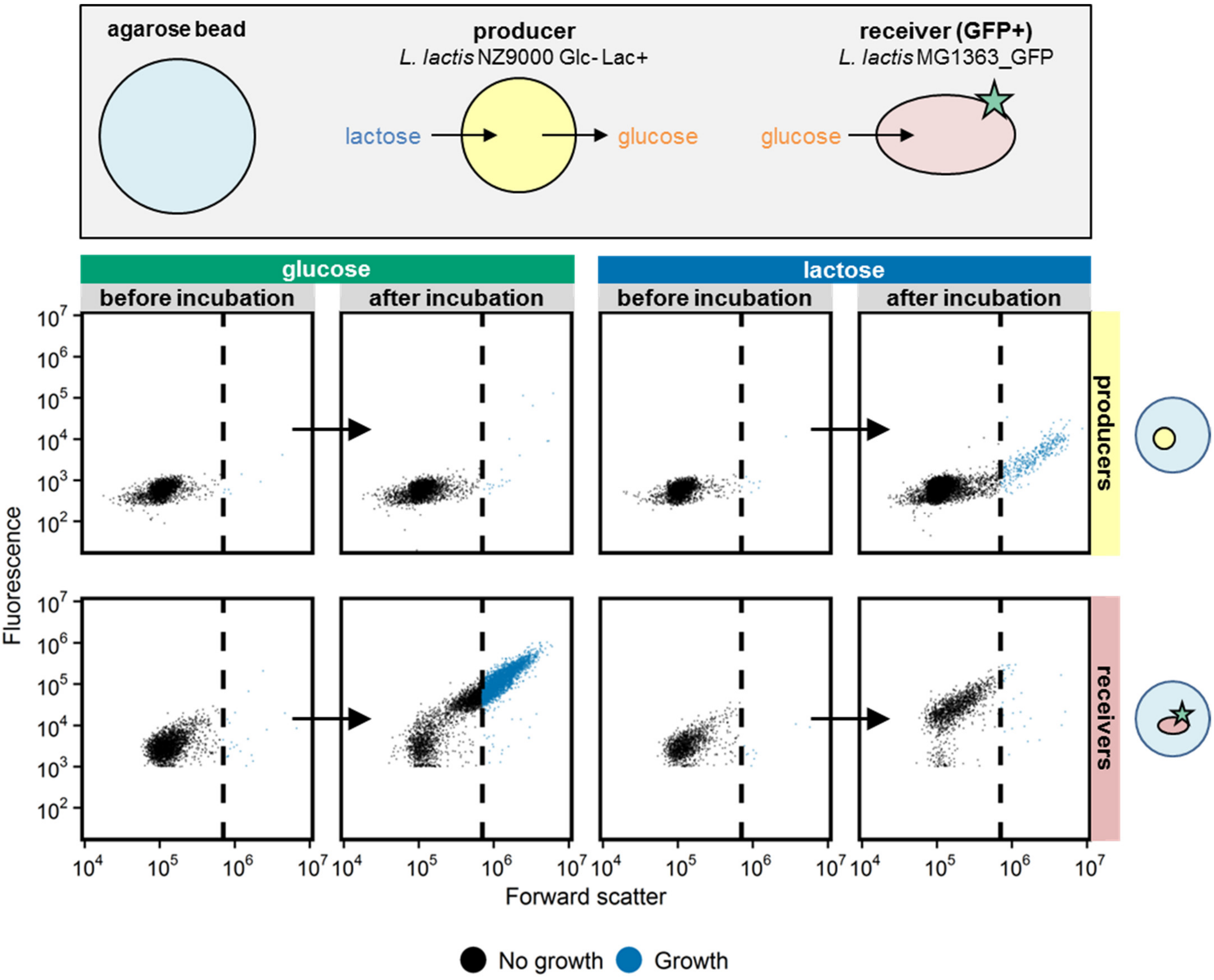

Figure S5. Flow cytometry gating strategy and data analysis. Agarose beads were inoculated with producer cells (top row) or receiver cells (bottom row). Both samples also contained empty agarose beads. The beads were incubated surrounded by medium with glucose (left) or lactose (right). Producer cells can only grow in the presence of lactose, receiver cells only in the presence of glucose (Figure 2A). Agarose beads were measured using flow cytometry before and after incubation. Flow cytometry data was gated according to Figure S4 step 1, and shows that the forward scatter increases only significantly when cells could grow.

Section 4: Reaction-diffusion model in COMSOL Multiphysics

4.1 Geometry of the agarose bead model

Figure S6 shows the geometry of agarose bead model.

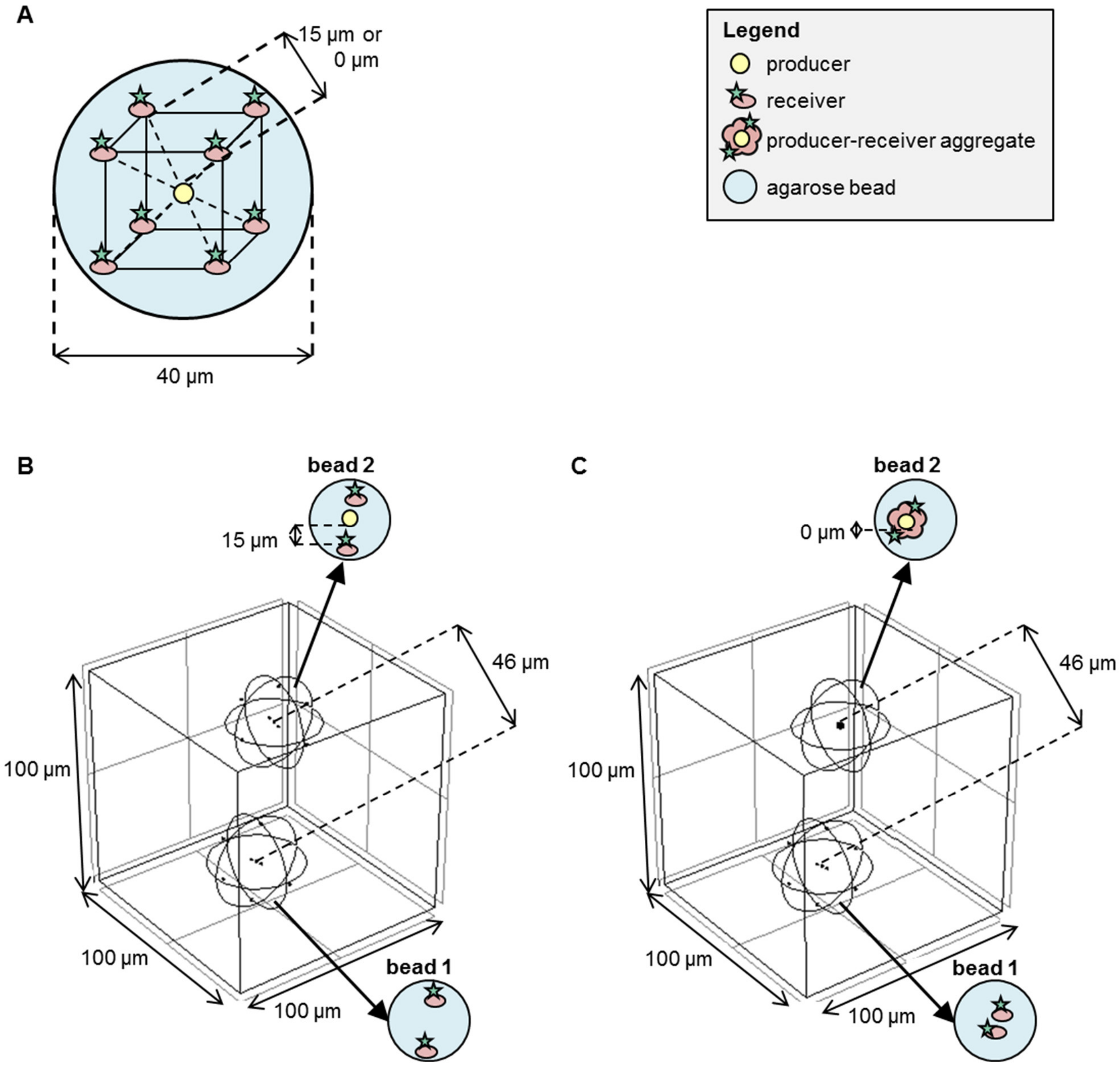

Figure S6: Model geometry implemented in COMSOL Multiphysics. (A) An agarose bead (sphere with diameter of 40 µm) contains 8 receiver cells and 0 or 1 producer cells. Each cell is a sphere with a diameter of 1 µm. Within the agarose bead receivers are placed at the virtual corners of a cube, and the producer in the middle. The distance between the surface of producers and receivers is either 0 or 15 µm. **(B and C)** An agarose bead without a producer (bead 1) and an agarose bead with one producer (bead 2) are placed in a cube of 100 µm (corresponding to $2 \cdot 10^6$ beads/mL). In (B) the distance between the producer and receiver is 15 µm, in (C) they are in contact (0 µm). Producers and receivers in contact are placed in a micro-colony (sphere with a diameter of 4 µm) with a reduced diffusion coefficient ($D_{eff,s}$).

4.2 Reaction-diffusion model and parameter values

Material balance. The spatial distribution (x,y,z) and change in time (t) of the concentration C_s (mol/m^3) of glucose in the bead and surrounding liquid resulted from solving the partial differential equation which balances the diffusion rate with a reaction rate r_s:

$$\frac{\partial C_s}{\partial t} = D_s\left(\frac{\partial^2 C_s}{\partial x^2} + \frac{\partial^2 C_s}{\partial y^2} + \frac{\partial^2 C_s}{\partial z^2}\right) + r_s$$

Diffusion. The same diffusion coefficient of glucose, D_s (m^2/s), was used outside and inside the agarose beads. It was set to the value of D_s in water [2], because D_s in agarose gels is similar to that in water [3]. The effective diffusion coefficient in micro-colonies depends on the void fraction, i.e. volume not occupied by cells per total micro-colony volume, and the tortuosity [4, 5]. Cells growing in an agarose matrix form dense colonies, therefore the effective diffusion coefficient within the colonies ($D_{eff,s}$) was set to 10% of the diffusion coefficient in water (D_s) [2, 6].

Reaction. The net glucose rate r_s (mol/m^3/s) results as the difference between production and consumption at a certain position in space, $r_s = q_pC_x - q_sC_x$. The specific glucose production rate (q_p) of *L. lactis* NZ9000 Glc-Lac+ is the same as its specific lactose uptake rate, as each lactose molecule contains one glucose molecule. The q_p was therefore set to a constant value of 1 molP/CmolX/h [7] and applied within the producer cells. Simulations which did include the lactose concentration and Monod kinetics for lactose consumption yielded similar results as simulations with a constant q_p, therefore we adopted the simpler constant rate. For receivers the glucose uptake was assumed with a saturation (Monod) kinetics, $q_s = q_s^{max} \cdot C_s/(K_s + C_s)$. We used the K_s of the highest affinity glucose transporter of *L. lactis* MG1363 [1], and q_s^{max} was set to 1 molS/CmolX/h [8]. To calculate the biomass concentration C_x (CmolX/m^3) we assumed a molecular weight of biomass of 24.6 grams per

Cmol dry biomass ($CH_{1.8}O_{0.5}N_{0.2}$) [9, 10], a cellular water content of 70 wt% [11] and a cellular density of 1000 g/L [11]. These values lead to a glucose production or maximal glucose consumption rate of 38 mol/m^3/s. A competing glucose-consumer was modelled by adding glucose consumption outside agarose beads with the same Monod kinetics as that of receivers. Cell growth was not incorporated in the model.

Boundaries. The concentration at the agarose bead surface was based on a partition coefficient which was set to 0 when incubation in oil was modelled, and to 1 for incubation in CDM. The liquid domain (cube) boundaries were insulated (no-flux boundary condition).

Parameters. Table S1 lists the default parameters used in the COMSOL Multiphysics model with sources for their values.

Table S1: Default parameter values of the COMSOL Multiphysics model

**Parameter**	**Symbol**	**Value**	**Source**
Maximum specific uptake rate of glucose	q_s^{max}	38 mol/m^3/s	[8]
Specific production rate of glucose	q_p	38 mol/m^3/s	[7]
Half-saturation (Monod) coefficient	K_s	0.01 mM	[1]
Diffusion coefficient in water and agarose beads	D_s	$6.7 \cdot 10^{-10}$ m^2/s	[2]
Effective diffusion coefficient in micro-colonies	$D_{eff,s}$	$0.10 \cdot D_s$	[2, 6]

4.3 Predicted concentration gradients in two- and three-dimensional systems

Table S2 summarizes the concentration gradient profiles from Figure 1.

Table S2. Effect of the diffusion coefficient on glucose concentration gradient profiles. Characteristics of the concentration gradient profiles from Figure 1. Values indicate the distance from producer cells at which the glucose concentration dropped below 10 μM.

**Diffusion coefficient of glucose (m^2/s)**			**No metabolite-sink**		**Metabolite-sink**	
			**2D**	**3D**	**2D**	**3D**
D_s	$6.7 \cdot 10^{-10}$	[2]	NA	NA	269 μm	0.7 μm
$D_{eff,biofilm,s}$	$0.25 \cdot D_s$	[2]	NA	NA	418 μm	3.2 μm
$D_{eff,colony,s}$	$0.10 \cdot D_s$	[2, 6]	NA	NA	454 μm	7.3 μm

4.4 Visualization of glucose concentrations and glucose production rates

Figure S7 shows the glucose concentration (Figure S7A) and glucose production rate (Figure S7B) as predicted by the model described in section 4.1 and 4.2. Profiles for aggregated cells and for cells 15 μm away from each other are shown.

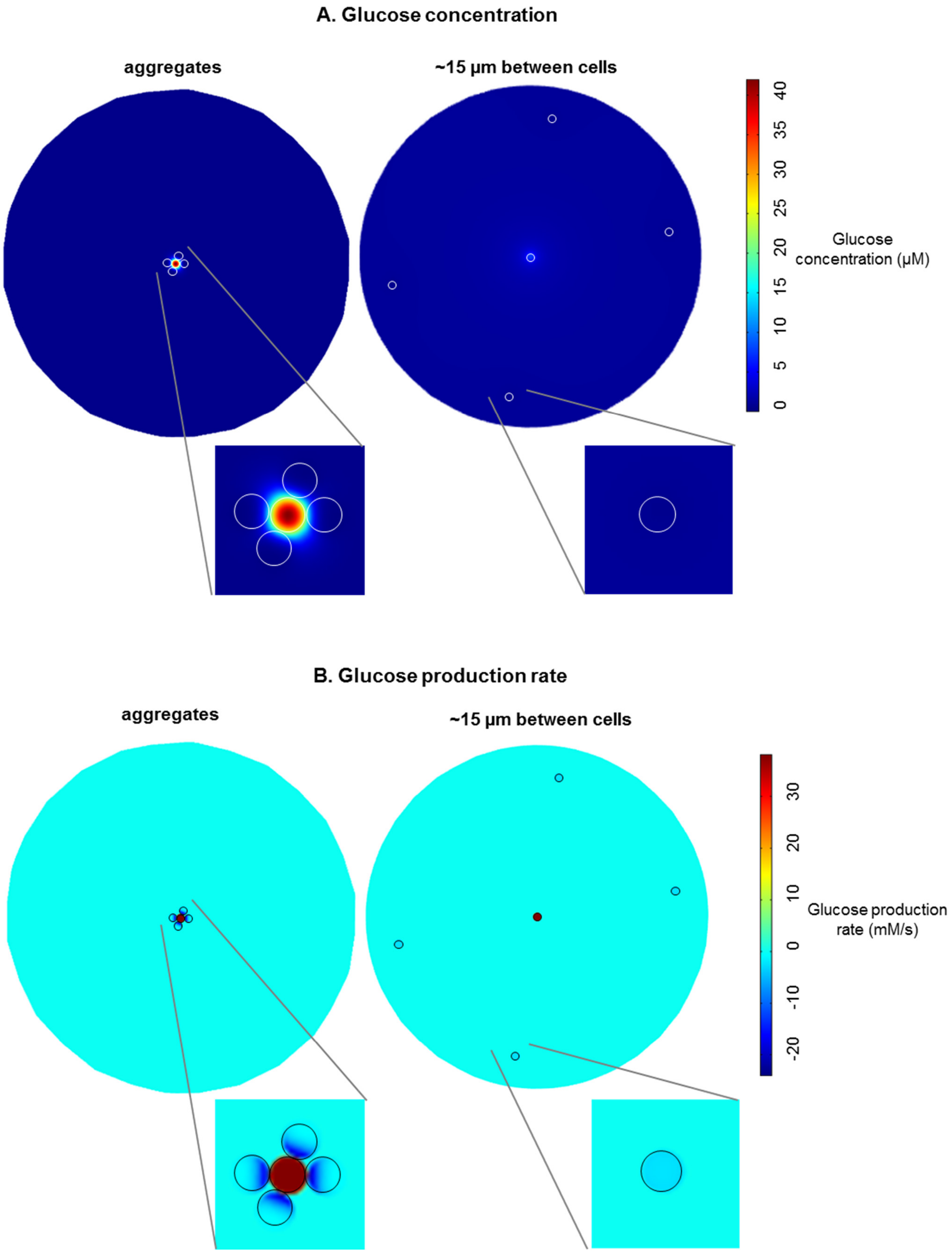

Figure S7: Predicted glucose concentration (A) and glucose production rates (B) in agarose beads surrounded by CDM. A time dependent study in COMSOL Multiphysics yielded concentration gradients at several moments. We here plotted the model predictions for 5 hours of

incubation in the presence of competing glucose-consumers, without considering growth of the cells. The glucose concentration **(A)** and the glucose production rate **(B)** after 5 hours are plotted over a plane crossing the producer cell and four of the eight receiver cells. Profiles for aggregated cells and for cells 15 μm away from each other are shown.

4.5 Factors affecting the interaction distance

Several factors affect the interaction distance between cells. We simulated the effect of the metabolite production rate (q_p), the maximal metabolite uptake rate (q_s^{max}), and differences in diffusion coefficients to represent aqueous conditions, biofilm and colony matrixes.

q_p and q_s^{max} variation in absence of a metabolite-sink

To analyze how the concentration gradient in absence of a metabolite-sink is affected by q_p and q_s^{max} we modelled a five-fold change in the glucose production rate (q_p) and/or the maximal glucose uptake rate (q_s^{max}) (Figure S8). Although a high q_p and/or a low q_s^{max} resulted in a higher minimal glucose concentration, predicted concentration gradients had a similar shape: receivers 0 or 15 μm from producers still saw similar glucose concentrations.

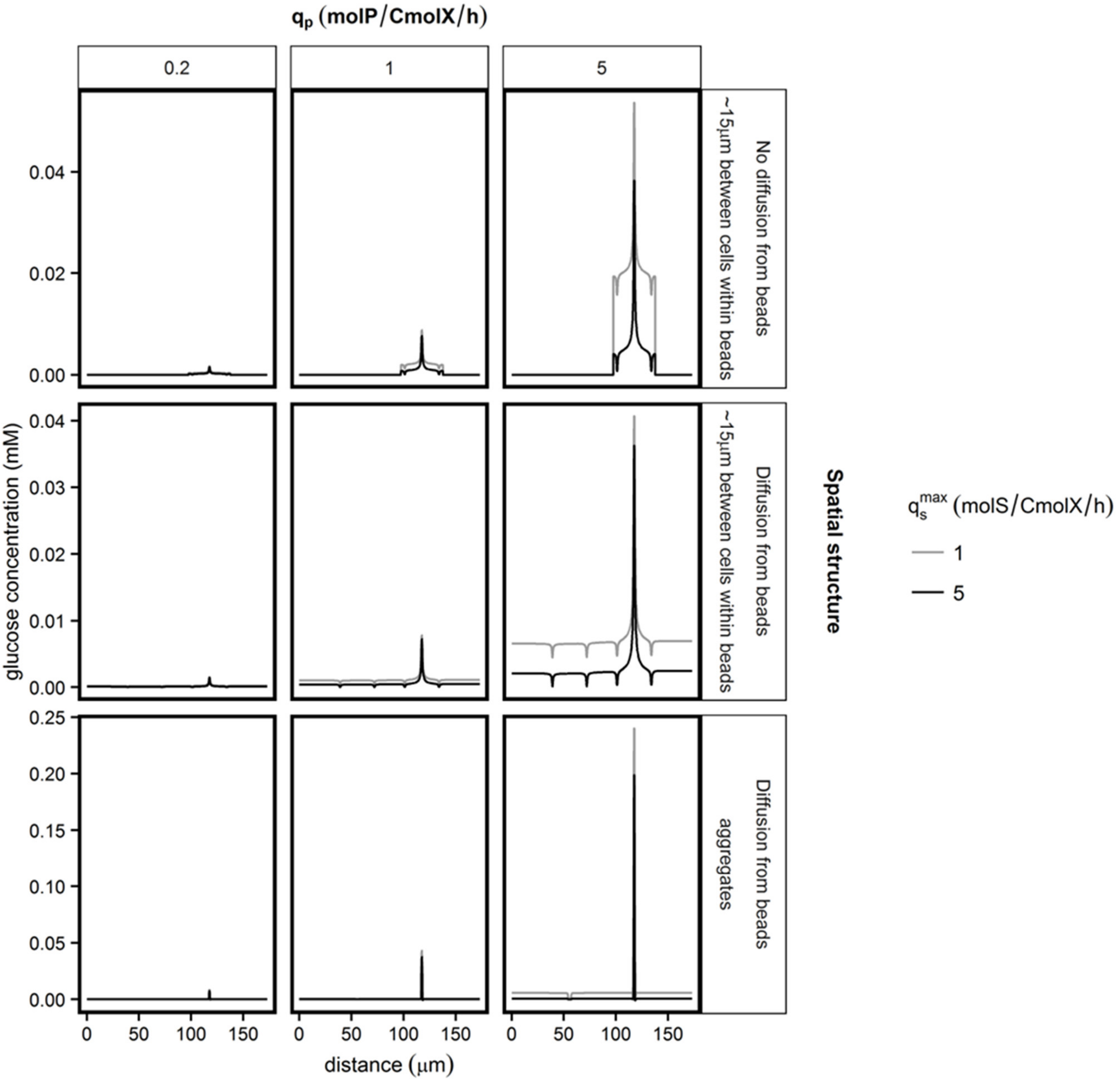

Figure S8: Predicted concentration gradients in absence of a metabolite-sink, after varying q_p and q_s^{max}. q_p and q_s^{max} were changed five-fold in absence of a metabolite-sink. A time dependent study in COMSOL Multiphysics yielded concentration gradients at several moments. This figure shows the predicted concentration gradients along the diagonal after 5 hours of incubation without considering growth of the cells, for different spatial structures.

q_p variation in the presence of a metabolite-sink

Figure S8 showed that an increase in q_p in absence of a metabolite-sink resulted in a higher minimal glucose concentration. This suggests that in the presence of a metabolite-sink an increase in q_p might increase the interaction-distance. We therefore modelled a five-fold change in q_p in the presence of a metabolite-sink, and predicted concentration gradients and glucose uptake by receivers (Figure S9). Figure S9A shows that an increase in q_p increases the width of the concentration gradient, resulting in an increased interaction distance. However, cells 15 μm from producers still see a very low glucose concentration and interactions are in the low μm-range, similar to what we observed in our experiments (Figure $3D_3$ and Figure $3D_4$). Figure S9C shows that when cells are randomly distributed within agarose beads the predicted glucose uptake can change. However, in over 90% of the simulated combinations the predicted glucose uptake changed less than four-fold compared to the default positioning of Figure 2B. As this observed change is small compared to the difference between separated and aggregated cells (Figure S9B), we used the default positioning in all other simulations.

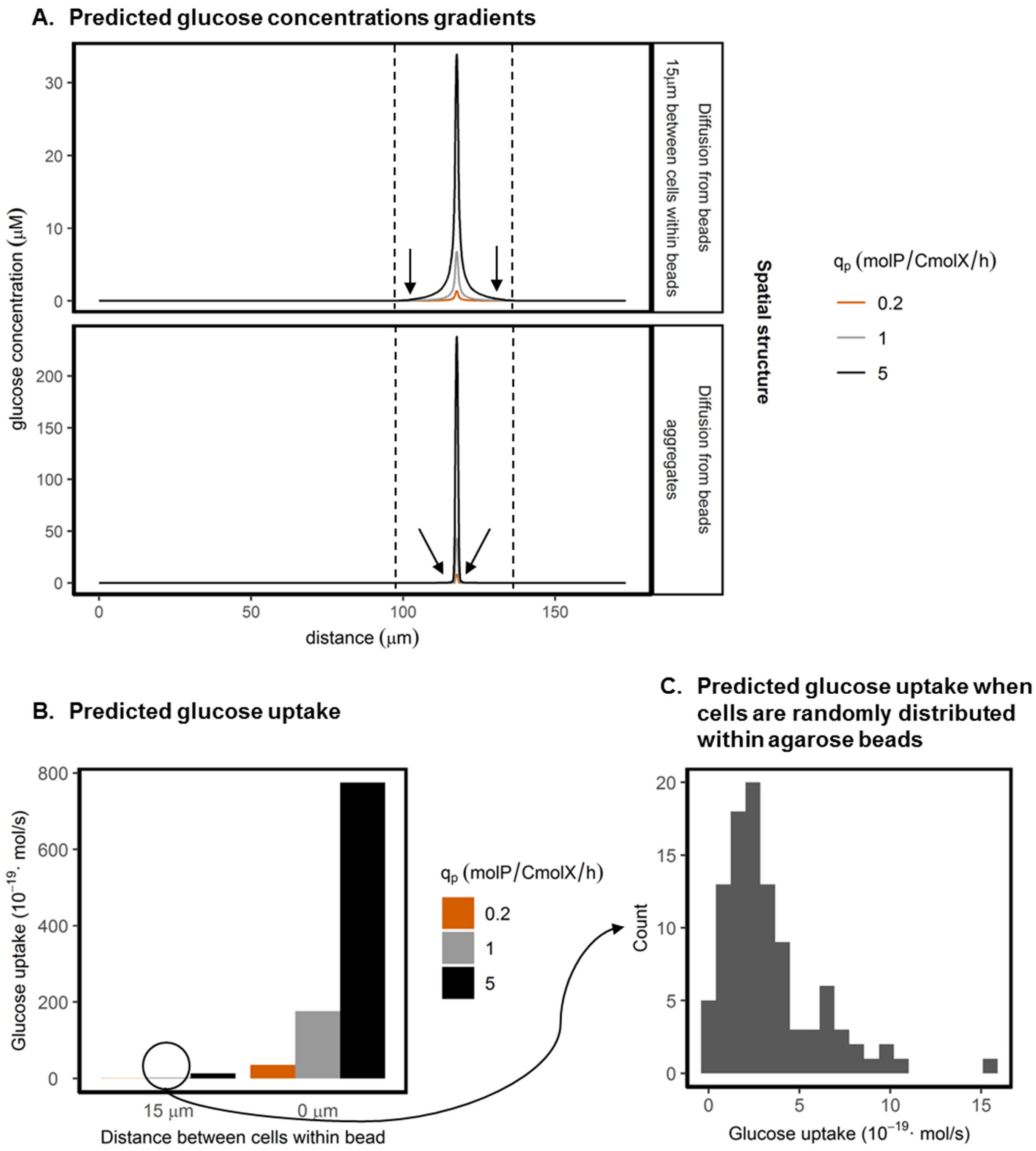

Figure S9: Predicted concentration gradients in the presence of a metabolite-sink for different q_p values. q_p was increased and decreased five-fold in the presence of a metabolite-sink. A time dependent study in COMSOL Multiphysics yielded concentration gradients at several moments. We here plotted the model predictions for 5 hours of incubation in the presence of competing glucose-consumers, without considering growth of the cells. **(A)** Predicted concentration gradients along the

diagonal in different spatial structures. Arrows indicate the location of receiver cells (default positioning, as shown in Figure 2B). Dashed lines indicate the border between the beads and the surrounding liquid containing the metabolite-sink. **(B)** Predicted glucose uptake of receivers in the conditions shown in panel (A). **(C)** For 15 μm between cells within beads and a q_p of 1 molP/CmolX/h, this panel shows the predicted glucose uptake when cells are randomly distributed within agarose beads. We simulated 100 configurations, with each 9 (1 for the producer cell and 8 for the receiver cells) randomly picked coordinates within the agarose bead.

Variation in the glucose uptake of receivers

In the presence of a metabolite-sink the minimal metabolite concentration is low, and we therefore hypothesized that the affinity of the receiver for the metabolite might affect the interaction distance between cells. We therefore analyzed the effect of the glucose affinity (K_s) and maximal uptake rate (V^{max}) of receivers on their ability to utilize the glucose made by the producer. To model the individual glucose transporter types of *L. lactis* MG1363 in COMSOL the K_m values of PTSman, PTScel and GlcU as reported by Castro *et al.* were used [1]. For *L. lactis* NZ9000_GFP_GlcU the q_s^{max} was reduced with a factor four, which reflects the lower V_{max} of this transporter [1]. We calculated the glucose uptake for the different mutants after 5 hours in the presence of competing glucose-consumers, without considering growth of the cells. The effective diffusion coefficient within aggregates ($D_{eff,s}$) varies from 10-70% of the diffusion coefficient in water, depending on the density of the micro-colony [2, 6]. Figure S10 shows the glucose uptake when $D_{eff,s}$ is 10%, 30% and 70%. We included a sensitivity analysis for five-fold changes in q_p and q_s^{max} values, which shows that at high q_p values the difference in glucose uptake of high and low affinity glucose transporters is lowest.

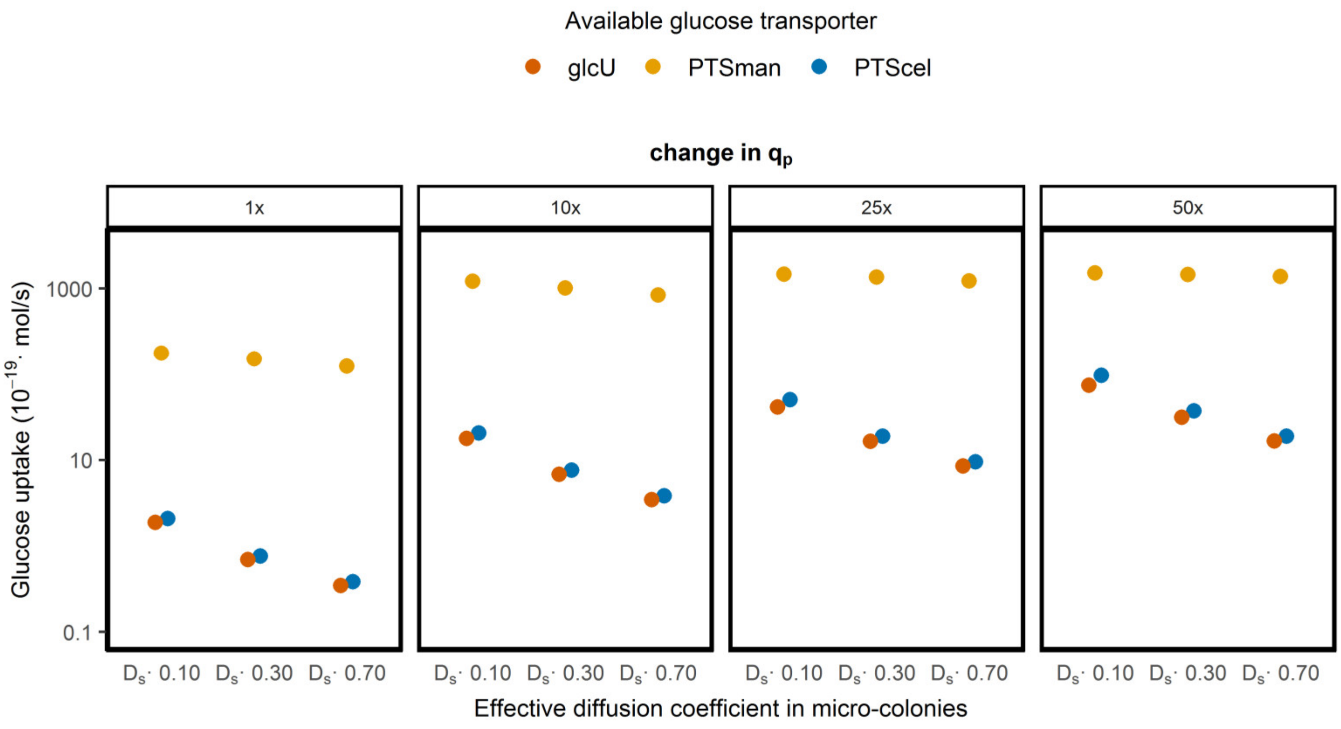

Figure S10: Predicted glucose uptake. We modelled receiver cells with different glucose affinities. A time dependent study in COMSOL Multiphysics yielded concentration gradients at several moments. We calculated the predicted glucose uptake by receivers after 5 hours of incubation in the presence of competing glucose-consumers, without considering growth of the cells. A sensitivity analysis for increasing q_p values and different effective diffusion coefficients within micro-colonies were incorporated.

Variation in the diffusion coefficient in the presence of a metabolite-sink

Factors like viscosity, the presence of extracellular polymeric substances and the local cell concentration affect the diffusion coefficient, and therefore the concentration gradient profiles of metabolites [2, 4]. We therefore used different diffusion coefficients to model diffusion in aqueous-, biofilm- and colony-like conditions (Figure S11). Figure S11 shows that an increase in the diffusion coefficient increases the glucose uptake from receivers 15 µm away from producers. However, the glucose uptake by receivers in producer-receiver aggregates is still significantly higher.

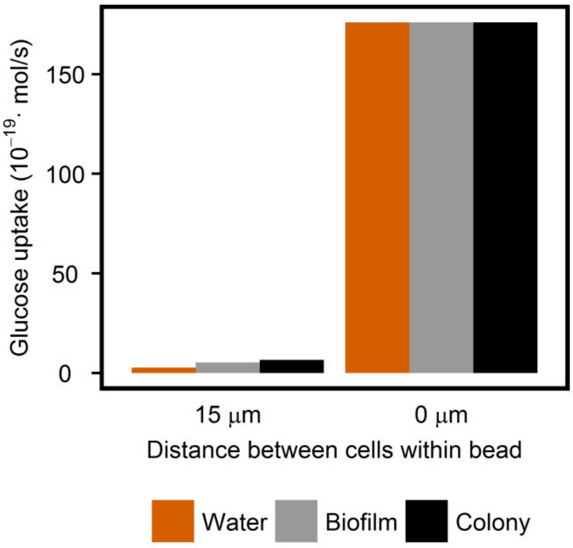

Figure S11: Effect of the diffusion coefficient on the glucose uptake of receiver cells. The diffusion coefficient of glucose in water (D_s) was set to $6.7 \cdot 10^{-10}$ m^2/s [2]. The effective diffusion coefficient of glucose representing biofilms ($D_{eff,biofilm,s}$) was set to 25% of D_s [2]. The effective diffusion coefficient of glucose representing a colony ($D_{eff,colony,s}$) was set to 10% of D_s [2, 6]. A time dependent study in COMSOL Multiphysics yielded concentration gradients at several moments. This figure shows the predicted glucose uptake by receivers after 5 hours of incubation in the presence of competing glucose-consumers, without considering growth of the cells.

Section 5: Growth rate determination

Strains were incubated in CDM + 0.2 wt% glucose in a 96-well plate. The OD_{600} was measured every six minutes for 24 hours using a SPECTRAmax 384 plus plate reader (Molecular Devices, San Jose, CA, USA). OD_{600} measurements were background corrected, ln-transformed and the slope of the region with exponential growth was calculated as the growth rate (Table S3).

Table S3. Growth rates of receivers with different glucose transporter types (n=22).

**Strain**	**Growth rate ± SD (h^{-1})**
L. lactis MG1363_GFP	1.25 ± 0.04
L. lactis NZ9000_GFP_GlcU	0.79 ± 0.04
L. lactis NZ9000_GFP_PTSman	0.74 ± 0.11
L. lactis NZ9000_GFP_PTScel	0.57 ± 0.03

Section 6: Flow cytometry data for receivers harboring only one glucose transporter type with varying affinities and V_{max}

We constitutively expressed GFP in three previously constructed *L. lactis* NZ9000 mutants with only one glucose transporter type - PTSman, PTScel and GlcU [1], and analyzed their growth in different spatial structures. This experiment focused on beads incubated in CDM (allowing glucose diffusion from beads), as we expected that under these conditions the transporter characteristics of receivers would be important. Figure S12A shows the experimental results when receivers were on average 15 µm from a producer within the same bead and incubated in CDM, whereas in Figure S12B the beads were incubated in medium with 10^9 glucose-consumers per mL as competing glucose-consumers. Figure S12C shows the experimental results of producer-receiver aggregates, incubated in CDM with 10^9 glucose-consumers per mL as competing glucose-consumers. Without competing glucose-consumers we observed growth of both receivers with and receivers without a producer in their bead (Figure S12A), while with competing glucose-consumers only producers could grow (Figure S12B). In producer-receiver aggregates receiver cells were able to grow, despite the presence of competing glucose-consumers (Figure S12C). The results were similar for all glucose transporter types, and were consistent with the results of the wildtype (Figure 3, Table 2).

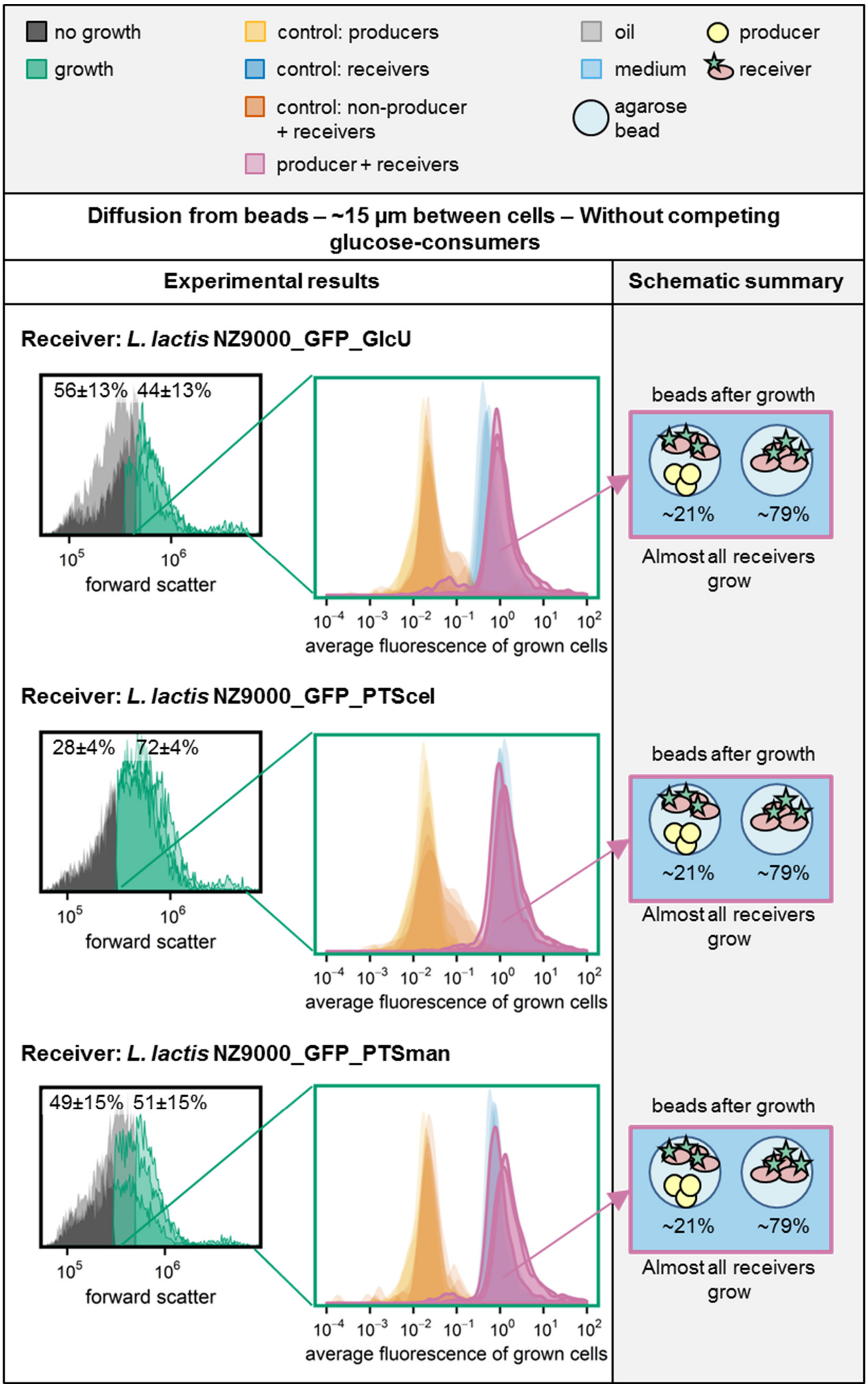

Figure S12A. Glucose accumulates, allowing receivers to grow independently of the available glucose transporter type. See the complete caption on page 28.

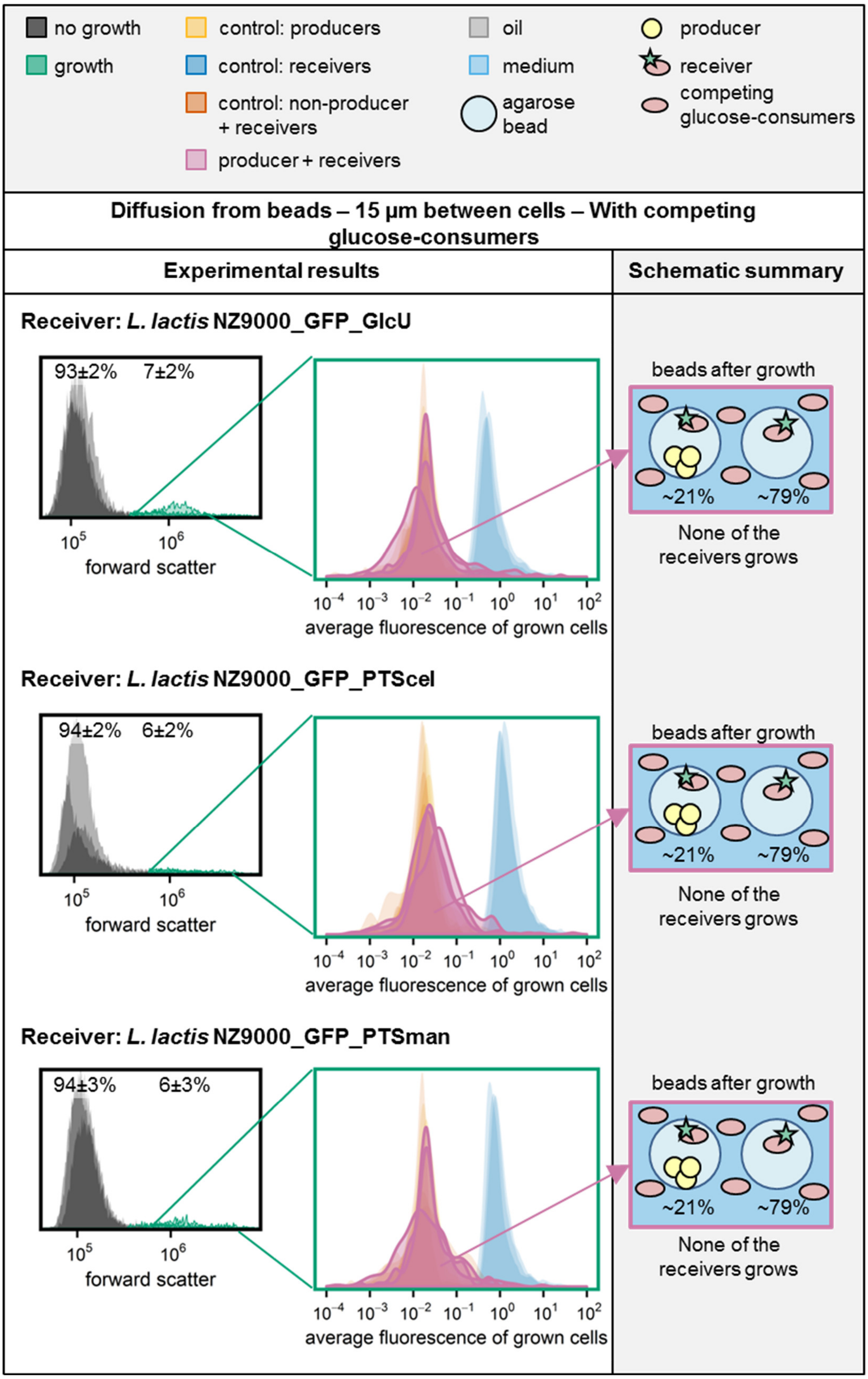

Figure S12B. In the presence of competing glucose-consumers receivers cannot grow, independently of the available glucose transporter type. See the complete caption on page 28.

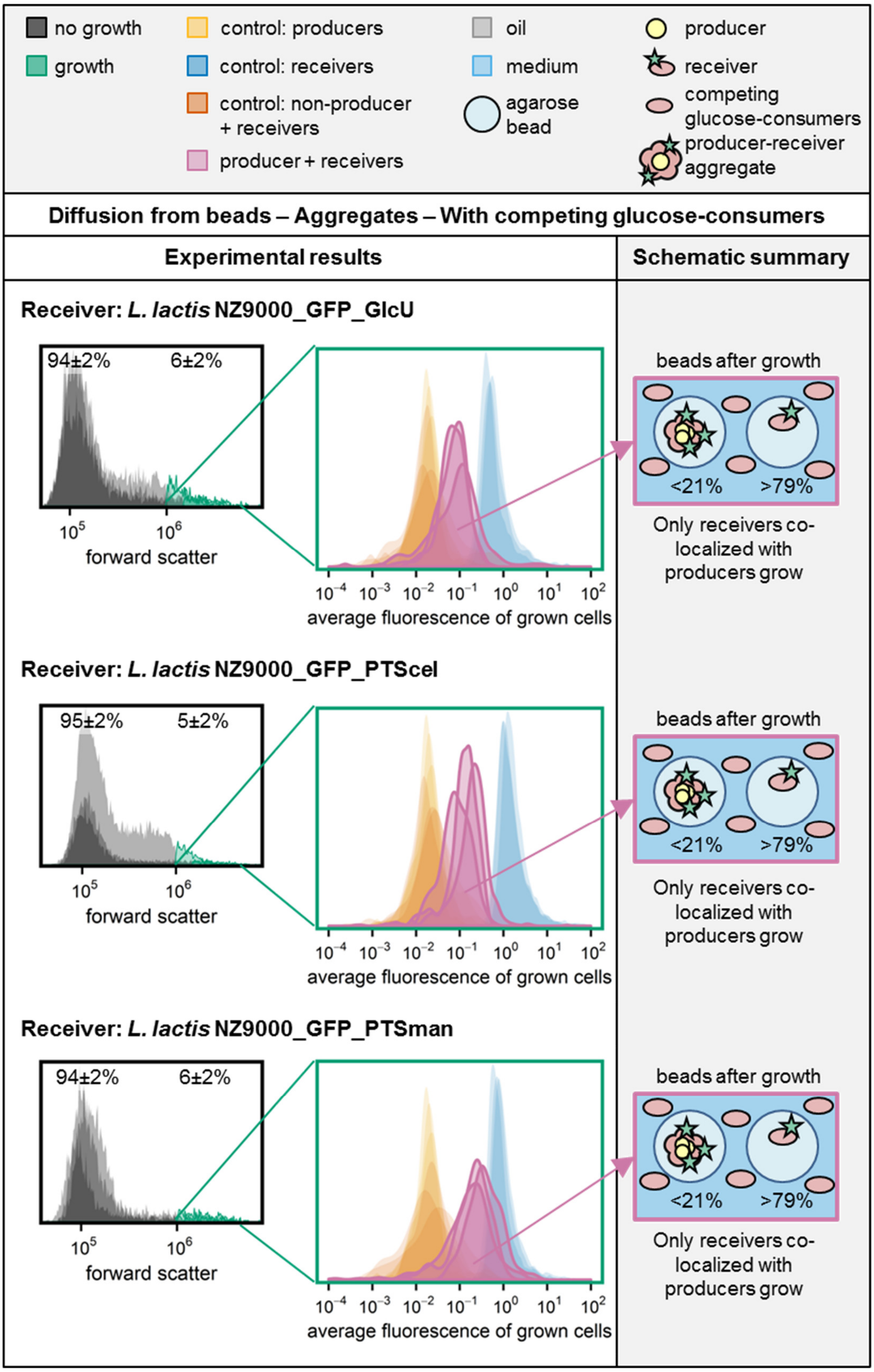

Figure S12C. Despite the presence of competing glucose-consumers receivers within producer-receiver aggregates can grow, independently of the available glucose transporter type. See the complete caption on page 28.

Figure S12: Response of consortia containing receivers with different glucose affinities. The density plots on the left indicate the percentage of beads that were gated as "growth" in the co-culture of producer and receivers (n=3, around 5500 agarose beads measured per replicate). The density plots on the right show the average fluorescence of the grown cells. This scales with the fraction of receiver cells in a bead, as shown by the control samples that are added in the same plot: receivers only, producers only and co-cultures of non-producers and receivers (n=3 for each of them). The non-producers and receivers, and the producers only controls are overlapping in all plots. The schematic drawing at the right summarizes the results from the presented density plots. **Different panels contain different spatial structures: (A)** Receivers that were on average 15 μm from a producer within the same bead, incubated in CDM. **(B)** Receivers that were on average 15 μm from a producer within the same bead, incubated in CDM with 10^9 glucose-consumers per mL as competing glucose-consumers. **(C)** Aggregates of producers and receivers, incubated in beads surrounded by CDM with 10^9 glucose-consumers per mL as competing glucose-consumers.

References

1. Castro R, Neves AR, Fonseca LL, Pool WA, Kok J, Kuipers OP, et al. Characterization of the individual glucose uptake systems of Lactococcus lactis: Mannose-PTS, cellobiose-PTS and the novel GlcU permease. *Mol Microbiol* 2009; **71**: 795–806.
2. Stewart PS. Diffusion in biofilms. *J Bacteriol* 2003; **185**: 1485–1491.
3. Weng L, Liang S, Zhang L, Zhang X, Xu J. Transport of glucose and poly(ethylene glycol)s in agarose gels studied by the refractive index method. *Macromolecules* 2005; **38**: 5236–5242.
4. Guélon T, Mathias J-DD, Deffuant G. Influence of spatial structure on effective nutrient diffusion in bacterial biofilms. *J Biol Phys* 2012; **38**: 573–588.
5. Dal Co A, van Vliet S, Kiviet DJ, Schlegel S, Ackermann M. Short-range interactions govern the dynamics and functions of microbial communities. *Nat Ecol Evol* 2020; **4**: 366–375.
6. Lawrence JR, Wolfaardt GM, Korber DR. Determination of diffusion coefficients in biofilms by confocal laser microscopy. *Appl Environ Microbiol* 1994; **60**: 1166–1173.
7. Boonmee M, Leksawasdi N, Bridge W, Rogers PL. Batch and continuous culture of Lactococcus lactis NZ133: experimental data and model development. *Biochem Eng J* 2003; **14**: 127–135.
8. Goel A, Eckhardt TH, Puri P, de Jong A, Branco dos Santos F, Giera M, et al. Protein costs do not explain evolution of metabolic strategies and regulation of ribosomal content: Does protein investment explain an anaerobic bacterial Crabtree effect? *Mol Microbiol* 2015; **97**: 77–92.
9. Heinzle E, Biwer AP, Cooney CL. Development of sustainable bioprocesses - modeling and assessment. 2006. John Wiley & Sons Ltd.

10. Oliveira AP, Nielsen J, Förster J. Modeling Lactococcus lactis using a genome-scale flux model. *BMC Microbiol* 2005; **5**.

11. Feijó Delgado F, Cermak N, Hecht VC, Son S, Li Y, Knudsen SM, et al. Intracellular water exchange for measuring the dry mass, water mass and changes in chemical composition of living cells. *PLoS One* 2013; **8**.
